# Protocol for the mWellcare trial: a multicentre, cluster randomised, 12-month, controlled trial to compare the effectiveness of mWellcare, an mHealth system for an integrated management of patients with hypertension and diabetes, versus enhanced usual care in India

**DOI:** 10.1136/bmjopen-2016-014851

**Published:** 2017-08-11

**Authors:** Dilip Jha, Priti Gupta, Vamadevan S Ajay, Devraj Jindal, Pablo Perel, David Prieto-Merino, Pramod Jacob, Jonathan Nyong, Vidya Venugopal, Kavita Singh, Shifalika Goenka, Ambuj Roy, Nikhil Tandon, Vikram Patel, Dorairaj Prabhakaran

**Affiliations:** 1 Centre for Control of Chronic Conditions, Public Health Foundation of India, Gurgaon, India; 2 Centre for Control of Chronic Conditions, London School of Hygiene and Tropical Medicine, London, UK; 3 Applied Statistical Methods in Medical Research Group, Universidad Catolica San Antonio de Murcia, Murcia, Spain; 4 Department of Epidemiology, University of Pittsburgh, Graduate School of Public Health, Pittsburgh, USA; 5 Department of Endocrinology and Metabolism, Centre for Control of Chronic Conditions, All India Institute of Medical Sciences, New Delhi, India; 6 Department of Cardiology, Centre for Control of Chronic Conditions, All India Institute of Medical Sciences, New Delhi, India; 7 Department of Global Health and Social Medicine, Harvard Medical School, Boston, USA

**Keywords:** hypertension, diabetes, mHealth, decision support system, integrated management of chronic conditions

## Abstract

**Introduction:**

Rising burden of cardiovascular disease (CVD) and diabetes is a major challenge to the health system in India. Innovative approaches such as mobile phone technology (mHealth) for electronic decision support in delivering evidence-based and integrated care for hypertension, diabetes and comorbid depression have potential to transform the primary healthcare system.

**Methods and analysis:**

mWellcare trial is a multicentre, cluster randomised controlled trial evaluating the clinical and cost-effectiveness of a mHealth system and nurse managed care for people with hypertension and diabetes in rural India. mWellcare system is an Android-based mobile application designed to generate algorithm-based clinical management prompts for treating hypertension and diabetes and also capable of storing health records, sending alerts and reminders for follow-up and adherence to medication. We recruited a total of 3702 participants from 40 Community Health Centres (CHCs), with ≥90 at each of the CHCs in the intervention and control (enhanced care) arms. The primary outcome is the difference in mean change (from baseline to 1 year) in systolic blood pressure and glycated haemoglobin (HbA1c) between the two treatment arms. The secondary outcomes are difference in mean change from baseline to 1 year in fasting plasma glucose, total cholesterol, predicted 10-year risk of CVD, depression, smoking behaviour, body mass index and alcohol use between the two treatment arms and cost-effectiveness.

**Ethics and dissemination:**

The study has been approved by the institutional Ethics Committees at Public Health Foundation of India and the London School of Hygiene and Tropical Medicine. Findings will be disseminated widely through peer-reviewed publications, conference presentations and other mechanisms.

**Trial registration:**

mWellcare trial is registered with Clinicaltrial.gov (Registration number NCT02480062; Pre-results) and Clinical Trial Registry of India (Registration number CTRI/2016/02/006641). The current version of the protocol is Version 2 dated 19 October 2015 and the study sponsor is Public Health Foundation of India, Gurgaon, India (www.phfi.org).

**Strengths and limitations of the study**

- mWellcare trial is the first study in India, assessing the effectiveness of mHealth technology for integrated management of hypertension and diabetes at primary care level in a public health setting.
- The study follows a cluster randomised controlled trial design.
- The study uses nurse for shifting/sharing some of the patient management tasks from physicians.
- The Intervention provides clinical decision support to physician for standardised and integrated management of hypertension and diabetes along with comorbid conditions such as depression and alcohol use disorder.
- The mWellcare intervention is being implemented in the real-world scenario aimed at using the existing human resource with an aim of scalability.
- Effectiveness of the trial will also depend on the availability of drugs recommended by the decision support system. Drugs are supplied by the state government and there are variations in availability across the state and districts, although such variations are likely to affect both arms equally.

## Background and rationale

Cardiovascular disease (CVD) and diabetes are the leading causes of premature (<60 years) adult deaths in India with projections indicating almost threefold increase to 18 million premature years of life lost by 2030, a greater loss of life than the combined projected burdens for China, Russia and the USA.^1 2^ CVD and diabetes will result in $2.32 trillion loss in national income in India between 2012 and 2030.^3^ Primary care is considered as the appropriate setting for the prevention and control of chronic conditions and it is being strengthened through the National Programme for Prevention and Control of cancer, diabetes, cardiovascular diseases and stroke (NPCDCS) in India.^2 4 5^

The telecom industry in India has grown exponentially over past 15 years, from under 3.6 million mobile phone subscribers in March 2001 to over a billion subscribers in December 2015.^6^ Mobile phones and other mobile technologies require less infrastructure than other eHealth systems, making them a promising investment for developing countries wanting to strengthen and transform their weak health systems, and to overcome healthcare worker shortages.^7–9^ Systematic reviews have demonstrated a lack of robust evaluations of the impact of mHealth.^10–14^ This lack of adequate evaluations led to need to provide robust evidence on the safety, benefits and associated cost-effectiveness of mHealth systems.^8 9 15 16^ Systematic review and meta-analysis of studies evaluating role of decision support system for prevention of CVD show paucity of well-designed studies on patient outcomes.^17^ In addition, there is a dearth of evidence on effect of task shifting/sharing strategies for CVD risk reduction and chronic disease management.^18 19^ Through mWellcare we propose to enhance the quality of hypertension and diabetes care at primary care level in India by using mobile phone technology that implements a clinical decision support system and electronic health records for the use of healthcare professionals. The effectiveness of the mHealth system will be tested on a range of clinical and process-of-care indicators.

## Methods and analysis

### Objective

The primary objective is to evaluate mWellcare system in two states in India over a 12-month period to determine its effectiveness on the management of patients with hypertension and/or diabetes based on their clinical outcomes (systolic blood pressure (SBP) and HbA1c) compared with an enhanced care arm using a cluster randomised study design.

Secondary objective is to assess the impact of the mWellcare system on change in cardiovascular risk factors (body mass index (BMI), alcohol use, tobacco use) and 10-year CVD risk score, mental health status, process outcomes and cost-effectiveness.

### Trial design

mWellcare is a cluster randomised controlled trial with equal allocation of participants between intervention and enhanced care arms.

### Study setting

Twenty clusters each from two states: one in the north and the other in the south India have been selected for the trial. Each cluster is a Community Health Centre (CHC) which caters to a rural population of 120 000 and serves as a referral centre for four Primary Health Centres (PHCs) in the formal healthcare delivery system.^20^ CHCs were selected from districts covered under the NPCDCS. Allocation of the CHCs into intervention and control arm is described in the section on randomisation.

### Inclusion criteria

Participants aged 30 years and above intending to reside in the catchment area of CHCs for at least next 12 months were eligible for the trial. Participants were included if they were diagnosed case of hypertension with blood pressure measuring ≥140/90 mm Hg or type 2 diabetes mellitus with fasting blood sugar ≥140 mg/dL or postprandial blood sugar ≥200 mg/dL and if they provided informed consent.

### Exclusion criteria

Pregnant women, patients with type 1 diabetes, patients requiring immediate referral to tertiary care due to accelerated hypertension or diabetic complications, patients with learning difficulties or vision and/or hearing impairments, patient suffering from malignancy or life-threatening disease with death probable in 4 years and patients not residing in the catchment area of the CHC were excluded.

### Intervention

In the intervention arm, nurse and physician are providing treatment and follow‐up to recruited participants using mWellcare system. mWellcare system is a tablet computer-based Android application that is designed to store the health records electronically, provides decision support recommendation tailored to the participant’s compliance and risk level, enables long-term monitoring and follow-up and sends SMS reminders (to take medication and follow-up visits) to patient. Development of intervention involved adapting existing clinical management guidelines to the local context, development and validation of clinical algorithm and pilot testing of mWellcare system. Nurses and physicians in the intervention arm were trained in the use of mWellcare system and also provided ‘refresher’ training on the clinical management guidelines for hypertension and diabetes.

In the intervention arm, besides baseline information, the nurse enters information on comorbid conditions: depression (using patient health questionnaire 9), alcohol use disorder (using alcohol use disorder identification test) and previous medication details into mWellcare system and generates and prints decision support recommendation (DSR) for the physician. The DSR printout consists of patient profile, diagnosed condition, comorbid conditions, previous and current medication and recommended treatment plan. It also provides lifestyle change recommendation and the date of the next follow-up visit. After review of the DSR, the physician may agree or suggest changes in the treatment plan which is recorded in the application. Nurse provides lifestyle advice brochure (in local language) and explains the same to each participant. Each participant will be followed up for 12 months. During this period, participants will receive SMS reminders for follow-up visits scheduled as per clinical algorithm. During follow-up visits, nurse enters relevant parameter to the patient’s clinical record and generates DSR printout for physician’s review. Each intervention site gets monthly reports on number of participants reporting for scheduled follow-up and average change in clinical parameters.

In the enhanced care arm, nurse and physicians were provided ‘refresher’ training on the clinical management guidelines for hypertension and diabetes. In addition, charts on management of these conditions were provided to the facilities for prominent display at the outpatient department. Physicians in the enhanced care arm provide the management plan based on their assessment of clinical parameters of the participants. Nurse provides lifestyle advice brochure (in local language) and explains the same to each participant. Follow-up in the enhanced care arm is based on the assessment of clinical parameters of the participants by the physician.

### Outcomes

#### Primary outcomes

- Difference in mean change (from baseline to 1 year) in SBP between the two treatment arms.
- Difference in mean change (from baseline to 1 year) glycated haemoglobin (HbA1c) between the two treatment arms.

#### Secondary outcomes include

- Difference in mean change (from baseline to 1 year) between the two treatment arms for fasting plasma glucose, total cholesterol and predicted 10-year risk of CVD using recalibrated Framingham risk score.
- Differences in risk factors such as depression/anxiety, smoking behaviour, BMI and alcohol use between the two treatment arms.
- Comparison of costs associated with delivering the mWellcare intervention arm with respect to enhanced care.

### Participant timeline

Enrolment and baseline assessment were done during the first visit, and the participants were asked to come back for blood sample collection (for HbA1C and total cholesterol tests) within 2 weeks. The intervention started on the first visit and will continue for 12 months. After 12 months, endline assessment will be done.

### Sample size calculation

The primary outcomes will be analysed as the average change of the biomarker (SBP) and glycated haemoglobin (HbA1c)) from baseline to 12-month follow-up in each participant. In each of the trial arms, there will be an average change and the effect of the intervention will be the difference of these averages.

A recruitment of 40 participants with hypertension per cluster in 40 clusters will yield over 98% power with a type I error of 5% to detect a mean difference of the change of 4 mm Hg in SBP, between the intervention and enhanced care arm assuming a 15 mm Hg SD of the changes in both arms and an intraclass correlation (ICC)=0.05.^21^ A number of 40 participants with diabetes per cluster will yield a power above 99% for detecting a true difference in average change of HbA1c between the two arms of 0.37%, assuming a 1.1% SD of the changes in both arms and an ICC=0.04.^22 23^

All calculations are based on the formula for comparison of two means in cluster randomised trials proposed by Hayes and Bennett and assume a 20% loss to follow-up.^24^

### Recruitment

For the two primary outcomes (SBP and HbA1c), we planned to recruit a total of 90 participants per site with a minimum of 45 participants with each conditions: hypertension and diabetes. We estimated that the recruitment could be completed in 2 months enrolling a minimum of two to three participants a day. However, some sites had lower recruitment rate for either of the two conditions. Therefore, the final strategy was to stop recruitment of participants with either condition after reaching 45, and then complete recruitment of patient with the other condition. A total of 3702 participants were recruited from 40 CHCs.

**Figure 1:**
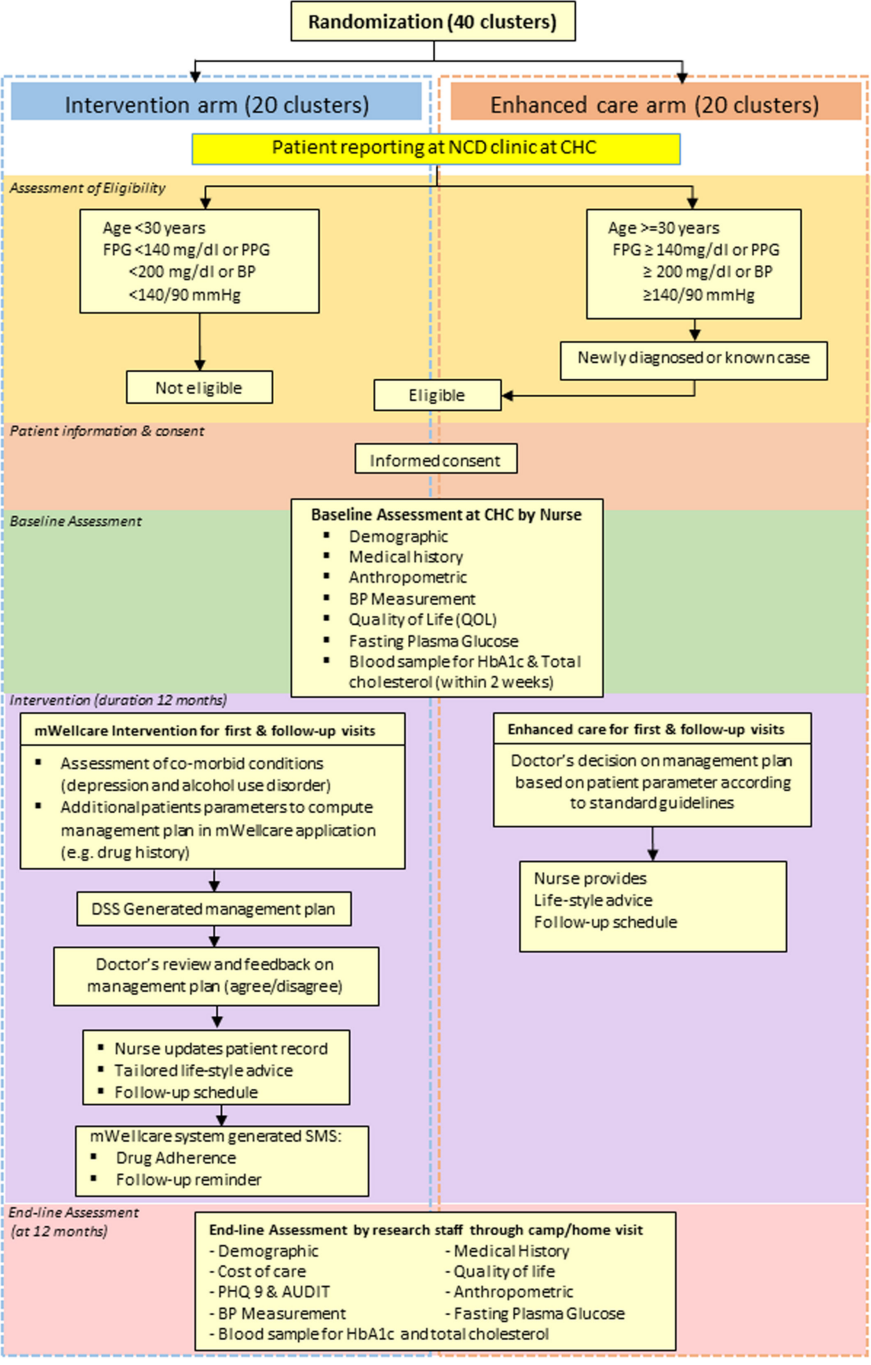
Trial flowchart. AUDIT, Alcohol Use Disorder Identification Test; BP, blood pressure; CHC, community health centre; FPG, fasting plasma glucose; NCD, non-communicable disease; PHQ9, patient health questionnaire; PPG, post-prandial glucose; SMS, short message service.

All individuals diagnosed to have hypertension and/or type 2 diabetes were assessed for eligibility by the nurse. All eligible participants were invited to participate in the trial. After providing written and verbal information about the trial, written consent was obtained by the nurse. The flow of trial participant is depicted in figure 1.

### Randomisation

Randomisation units are clusters (CHCs). The randomisation list was generated by a statistician independent of the trial using STATA SE V. 12. The list was stratified by states (Haryana and Karnataka) and within states by availability/non-availability of nurses recruited under NPCDCS. In Karnataka, stratification based on availability of nurses was done allocating 10 clusters in each category (nurse available under NPCDCS/not available). In Haryana, as there was no nurses recruited under NPCDCS, further stratification was not done. Clusters (CHCs) within each strata were randomised to intervention or enhanced care arm using block randomisation (with a block size of 2). Being a cluster randomised trial, only limited blinding could be enforced. The study statistician will remain blinded throughout the study until database is locked and study unblinded.

### Data collection, management and analysis

#### Baseline assessment

After consent, the nurse in both arms collected baseline data for the following parameters using a tablet computer: age, gender, marital status, occupation, alcohol use, smoking behaviour, health-related quality of life measured using EuroQol five dimensions questionnaire (EQ-5D), height, weight, blood pressure and blood sugar. Consented participants were asked to return on a scheduled day for blood sample collection for HbA1c and total cholesterol.

#### Endline assessment

Endline assessment of outcomes will be undertaken after completion of 12-month follow-up. The assessments will be carried out by independent outcome assessors inviting the participants to camps and mop up will be organised through home visits.

#### Data management

This study has only electronic version of the study forms and the dataset automatically synchronises with the server. There are built‐in data checks and further data cleaning will be done by trained data managers at Public Health Foundation of India (PHFI), under the supervision of the Principal Investigator. All electronic data are encrypted, password protected and stored in secure computer networks.

Data are monitored centrally for its quality and completeness using electronic validation and on-site monitoring. Recruitment and retention of participants was assessed by examining the number eligible for recruitment, number enrolled in the study and dropout from regular follow-up will be assessed at the completion of 12 months. In addition, we are carrying out central statistical monitoring (CSM) all through the trial. CSM aims to detect outliers and inliers, deviation of any cluster from the average and to detect potential data error or incorrect data collection procedure. Field coordinators undertake monthly visits to all sites, record their observations using Objective Systematic Structured Observations (OSSO) checklist regarding intervention delivery, source documents examination, protocol adherence and adverse event recording and reporting. The monthly visit reports are sent online to the research coordinating centre. In addition, study investigators undertook site visits to monitor enrolment process, intervention delivery and protocol adherence and reported to the trial management committee.

#### Statistical methods

Quantitative data analysis will be performed using STATA V. 13.0 (Stata). A statistical data analysis plan will be prepared and discussed with Data Safety Monitoring Board (DSMB) before data collection is completed.

The primary analyses will be conducted under the principle of intention to treat. All randomised participants will be analysed in the groups to which they were originally allocated to, regardless of whether they actually retained that specific group membership over the course of the trial or not. Participants who withdrew consent for use of their data will not be included in any analyses.

Demographic and clinical characteristics will be examined for participants at each CHC. Summaries will be presented as means and SD of those variables that are approximately normally distributed, or medians and IQRs for skewed variables. Categorical variables will be summarised as frequencies and percentages. Transformations will be used when distributional assumptions are not fulfilled for inferential tests on a continuous measure. If we find a considerable imbalance in some variables at baseline, we will consider adjusting for these variables by including them in the model to estimate the effect of the intervention. In principle, in a trial with individual blinded randomisation, there should be no major baseline differences between arms, but this may not be the case in cluster sample given that the number of samples are small, individuals are not completely blind. So there is some potential for baseline differences between individuals recruited in each arm and this will have to be adjusted for in the analysis.

We will also account for the influence of clustering at the CHC level on the outcome. Data will be analysed at the individual level and comparisons will be made between the mean changes in the primary outcomes in the intervention and control groups. Linear-mixed effect models adjusting for baseline measurements will be used with a random intercept for the CHC level to account for the clustering in the data.

Reporting of results will be in accordance with the principles of Consolidated Standards of Reporting Trials(CONSORT) statements extended for cluster randomised controlled trial (RCT).

#### Process evaluation

REAIM (reach, effectiveness, adoption, implementation and maintenance) framework for the process evaluation will be used for mWellcare trial.^25^ This would entail a combination of qualitative (observations, interviews, focus group discussions) and quantitative (open-ended and close-ended questionnaires) assessment.

Fidelity will be assessed through logs of training, indicators from OSSO checklist and data captured through mWellcare system. Besides reports of monitoring visits by experts will form a part of the assessment.

#### Economic evaluation

To help inform potential financing and adoption of the mWellcare intervention, compared with enhanced care (control), we will estimate the value for different stakeholders: patients (mean annual health expenditures in each arm), healthcare providers (incremental costs to deliver the intervention and cost-effectiveness: eg, incremental cost to prevent one CVD event) and society (cost-utility: eg, cost per quality-adjusted life years gained).

We will collect patient-level healthcare utilisation and medical costs data incurred during the trial regarding outpatient visits, diagnostic services, medications, hospitalisations and lost productivity.^26^ We will identify intervention costs from the non-communicable disease clinic and study expenditures (eg, costs related to accessing the mWellcare system, and sending the regular SMS updates, training costs, labour costs for nurses, physician’s time cost and overhead costs minus costs attributable only to research activities (eg, annual study visit)). We will compute the incremental costs of intervention (implementation, adverse effects, medical care associated) compared with control. For effectiveness measures, we will use between-group differences in SBP and HbA1c reductions. Utility data will be collected using the EQ5D Visual Analogue Scale.

If the primary clinical outcomes (SBP and HbA1c) are shown to differ substantially, a full economic evaluation of the lifetime costs, benefits and cost-effectiveness (in life years gained and quality-adjusted life years gained) of switching from usual care to mWellcare intervention using decision models will be performed.^27^ Uncertainty around incremental cost-effectiveness ratio will be estimated using non-parametric bootstrap methods. To inform societal perspective, we will calculate an incremental cost-utility ratio($\frac{costs_{intervention}-costs_{control}}{utility_{intervention}-utility_{control}}$).^28^ To standardise for the time value of money, we will use a 3% annual discount rate. Costs will be expressed in rupees and US$ (2016 value). We will conduct sensitivity analyses where discount rates, intervention costs, effectiveness and other model parameters will be varied to estimate cost-utility under different scenarios.^29^ We will also generate cost-effectiveness acceptability curve to demonstrate the probability of cost-effectiveness of the intervention at a range of willingness to pay threshold values. If no significant difference is found for the primary clinical outcomes, a cost-minimisation analysis will be performed.

#### Data monitoring

A four-member DSMB has been established. DSMB members are independent from sponsor and have no conflict of interest. The board members include experts in biostatistics, ethics and cardiovascular disease management. Based on observed beneficial or observed effect, DSMB will make recommendation to the Research Steering Group (RSG) in relation to the conduct of the trial (continue, change or terminate). The RSG takes responsibility for the design, conduct and analysis of the clinical trial. RSG is a multidisciplinary group who, collectively, has the scientific, medical and clinical trial management experience to conduct and evaluate the trial. In addition, a Research Management Committee) consisting of principal investigators, co-investigators, project manager and other team members have been formed to monitor progress of the study.

#### Serious adverse events

Occurrence of serious adverse events that include severe hypoglycaemia, CVD events, diabetes-related gangrene or amputation, end-stage renal disease and death is being monitored. Information about the occurrence of any adverse event is sought at all scheduled visits, and outside of scheduled visits by participant self‐report and tracking of non‐study-related visits. All events reported in the study will be duly notified to the overviewing ethics committees and DSMB in the annual progress report.

## Discussion

mWellcare is the first study in India, assessing the effectiveness of mHealth technology for integrated management of hypertension and diabetes at primary care level in a public health setting. Few trials have evaluated the impact of DSS on managing patients with hypertension and diabetes. A recent cluster randomised trial in Andhra Pradesh, India, which evaluated the use of DSS in hypertension management, demonstrated significant reduction in SBP among patient in primary healthcare facilities.^21^ However, this trial focused only on hypertension while the other cardiometabolic risk factors were not covered. The recently published results of Cardiometabolic Risk Reduction in South Asia (CARRS) Diabetes Trial shows significant improvement in diabetes care targets and cardiometabolic risk reduction among patients who underwent multicomponent quality improvement intervention comprising non-physician care coordinators and decision support electronic health records.^30^ However, the findings of CARRS trial are confined to urban specialist care settings.

mWellcare trial with its cluster randomised design will extend the evidence on impact of mHealth intervention on patient’s CVD outcomes at primary care level. It will provide evidence on the use of nurse for patient assessment and long-term follow-up using the mWellcare system thereby shifting/sharing some of the patient management tasks from physicians. It is also designed to provide decision support to physician for standardised and integrated management of hypertension and diabetes along with comorbid condition such as depression and alcohol use disorder.

The mWellcare intervention is being implemented in the real-world scenario aimed at using the existing human resource (as the nurse appointed at the outpatient clinics through the NPCDCS). The trial also incorporates cost-effectiveness analysis of the intervention. All these factors would be helpful in informing decision makers in allocating resources and scaling up the intervention based on the trial findings.

There are a few limitations for this study. The effectiveness of the trial will also depend on the availability of drugs, recommended by the decision support system, at the CHCs. Drugs are supplied by the state government and there are variations in availability across the state and districts, although such variations are likely to affect both arms equally. Another limitations is that the mWellcare system will be used only for the people who have been already diagnosed with hypertension or diabetes by the physician, and will not aid in opportunistic screening of patients coming to the CHC. This was done to keep both intervention and control arms at par. In case the intervention is scaled up, a screening module can be added to the mWellcare system.

### Conclusion

The mWellcare trial will provide evidence on effectiveness of the nurse-based mHealth intervention for integrated management of hypertension and diabetes at primary care level in India. Results from the trial will have direct policy relevance in adopting mHealth solution for managing CVDs at primary care level in India.

## Ethics and dissemination

mWellcare trial protocol and study documents have been approved by the Institutional Ethics Committee at PHFI and London School of Hygiene and Tropical Medicine. All information collected as part of this study will be kept strictly confidential. Personal identifiers will be removed before transferring data for analysis. Participant’s identity will be anonymous and postanalysis of the data, the tapes and transcripts will be destroyed. Participant’s name or identity will not be revealed in any of the publications arising from this research.

Findings from this study will be submitted for publication in peer-reviewed journals. The study results will be shared with health professionals (primary care providers), local government and decision makers as brief policy notes. The study investigators will also disseminate findings through professional conferences targeting primary and secondary care physicians, research community and public health policymakers more widely. The results of this study will provide policy-relevant recommendations for the uptake of mHealth interventions in the management of hypertension and diabetes in India.

At the end of the study, the investigators will form a Publication, Presentation and Ancillary Studies (PP&A) subcommittee, which will develop a suitable policy protecting the rights of involved organisations regarding ownership of study materials and data. The full editorial control will reside with the PP&A committee. All investigators will be given access to the cleaned data sets.

## Supplementary Material

Reviewer comments

Author's manuscript
