# Supplementary material for: Protocol for the mWellcare trial: a multicentre, cluster randomised, 12-month, controlled trial to compare the effectiveness of mWellcare, an mHealth system for an integrated management of patients with hypertension and diabetes, versus enhanced usual care in India
Source: BMJ Open. 2017 Aug 11;7(8):e014851. doi: 10.1136/bmjopen-2016-014851 (PMC5724108; doi:10.1136/bmjopen-2016-014851)
Supplement: Author's manuscript [file bmjopen-2016-014851.draft_revisions.pdf]

BMJ Open

mWellcare Trial: A multi-centre, cluster randomized, 12-month, controlled trial to compare the effectiveness of mWellcare, an mHealth system for an integrated management of patients with hypertension and diabetes, versus enhanced-usual care in India

Journal:	*BMJ Open*
Manuscript ID	bmjopen-2016-014851
Article Type:	Protocol
Date Submitted by the Author:	03-Nov-2016

Complete List of Authors:	Jha, Dilip; Public Health Foundation of India,<br>Gupta, Priti; Public Health Foundation of India<br>Vamadevan, Ajay; Public Health Foundation of India<br>Jindal, Devraj; Public Health Foundation of India<br>Perel, Pablo; LSHTM, EPH<br>Prieto-Merino, David; London School of Hygiene & Tropical Medicine<br>Jacob, Pramod; Public Health Foundation of India<br>Nyong, Jonathan; LSHTM, EPH<br>Venugopal, Vidya; University of Pittsburgh Graduate School of Public Health, Epidemiology<br>Singh, Kavita; All India Institute of Medical Sciences, Endocrinology & Metabolism<br>Goenka, Shifalika; Public Health Foundation of India<br>Roy, Ambuj; All India Institute of Medical Sciences, Cardiology<br>Tandon, Nikhil; All India Institute of Medical Sciences,<br>Patel, Vikram; London School of Hygiene and Tropical Medicine<br>Prabhakaran, Dorairaj; Centre for Chronic Disease Control, ; Public Health Foundation of India,

**Primary Subject Heading**:	Cardiovascular medicine
Secondary Subject Heading:	Diabetes and endocrinology, Evidence based practice, Public health
Keywords:	Hypertension < CARDIOLOGY, mHealth, Type 2 Diabetes Mellitus, Decision Support System, Task shifting, Integrated management of chronic conditions

SCHOLARONE™ Manuscripts

Title

mWellcare Trial: A multi-centre, cluster randomized, 12-month, controlled trial to compare the effectiveness of mWellcare, an mHealth system for an integrated management of patients with hypertension and diabetes, versus enhanced-usual care in India

Author:

Dilip Jha, Priti Gupta, Vamadevan S Ajay, Devraj Jindal, Pablo Perel, David Prieto-Merino, Pramod Jacob, Jonathan Nyong, Vidya Venugopal, Kavita Singh, Shifalika Goenka, Ambuj Roy, Nikhil Tandon, Vikram Patel, Dorairaj Prabhakaran

Corresponding author:
Name - Prof Dorairaj Prabhakaran
Postal Address - Public Health Foundation of India (PHFI)
4th Floor, Plot - 47, Sector – 44
Gurgaon – 122 002 (Haryana) India
Email – dprabhakaran@ccdcindia.org
Telephone: +91-124-4781400
Fax: +91-124-4781601

Full name, department, institution, city and country of all co-authors.

Dilip Jha
Senior Research Project Manager
Centre for Control of Chronic Conditions
Public Health Foundation of India, Gurgaon, India

Priti Gupta
Senior Research Associate
Centre for Control of Chronic Conditions
Public Health Foundation of India, Gurgaon, India

Vamadevan S Ajay
Senior Research Scientist
Centre for Control of Chronic Conditions
Public Health Foundation of India, Gurgaon, India

Devraj Jindal
Project Manager
Centre for Control of Chronic Conditions
Public Health Foundation of India, Gurgaon, India

Pablo Perel
Associate Professor
Centre for Control of Chronic Conditions
London School of Hygiene & Tropical Medicine, London, UK

David Prieto-Merino
Associate Professor,
Centre for Control of Chronic Conditions
London School of Hygiene & Tropical Medicine, London, UK

For peer review only

Pramod Jacob
Health Informatics Consultant
Centre for Control of Chronic Conditions
Public Health Foundation of India, Gurgaon, India

Jonathan Nyong,
Research Fellow
Centre for Control of Chronic Conditions
London School of Hygiene & Tropical Medicine, London, UK

Vidya Venugopal
Postdoctoral Associate
Department of Epidemiology
University of Pittsburgh Graduate School of Public Health, Pittsburgh, USA

Kavita Singh
PhD Student
Centre for Control of Chronic Conditions
All India Institute of Medical Science, New Delhi, India

Shifalika Goenka,
Additional Professor
Centre for Control of Chronic Conditions
Public Health Foundation of India, Gurgaon, India

Ambuj Roy
Additional Professor, Department of Cardiology
Centre for Control of Chronic Conditions
All India Institute of Medical Science, New Delhi, India

Nikhil Tandon
Professor& Head, Department of Endocrinology & Metabolism,
Centre for Control of Chronic Conditions
All India Institute of Medical Science, New Delhi, India

Vikram Patel,
Professor
Centre for Control of Chronic Conditions
London School of Hygiene & Tropical Medicine, London, UK

Dorairaj Prabhakaran
Professor
Centre for Control of Chronic Conditions
Public Health Foundation of India, Gurgaon, India

Word count excluding title page, abstract, references, figures and tables: 4856

Keywords
Hypertension, diabetes, mHealth, decision support system, integrated management of chronic conditions

For peer review only - http://bmjopen.bmj.com/site/about/guidelines.xhtml

Abstract

Introduction

Rising burden of cardiovascular disease and diabetes is a major challenge to the health system in India. Innovative approaches such as mobile phone technology (mHealth) for electronic decision support in delivering evidence based and integrated care for hypertension, diabetes and comorbid depression has potential to transform the primary healthcare system.

Methods and analysis

mWellcare trial is a multi-centre, cluster randomized controlled trial evaluating the clinical and cost effectiveness of a mHealth system and nurse managed care for people with hypertension and diabetes in rural India. mWellcare system is an android-based mobile application designed to generate algorithm based clinical management prompts for treating hypertension and diabetes and also capable of storing health records, sending alerts and reminders for follow-up and adherence to medication. A total of 3600 participants will be recruited from 40 Community Health Centres (CHCs); 90 at each of the CHCs in the intervention and control (enhanced care) arms. The primary outcome is the difference in mean change (from baseline to one year) in systolic blood pressure and glycated haemoglobin (HbA1c) between the two treatment arms. The secondary outcomes are difference in mean change from baseline to one year in fasting plasma glucose (FPG), total cholesterol, predicted 10 year risk of Cardio-vascular disease (CVD), depression, smoking behavior, body mass index (BMI) and alcohol use between the two treatment arms and cost effectiveness.

Ethics and dissemination

The study has been approved by the institutional Ethics Committees at Public Health Foundation of India and the London School of Hygiene and Tropical Medicine. Findings will be disseminated widely through peer reviewed publications, conference presentations and other mechanisms.

Strength & limitations of the study

Strength:

- mWellcare trial is the first study in India, assessing the effectiveness of mHealth technology for integrated management of hypertension and diabetes at primary care level in a public health setting.
- The study follows a cluster randomised controlled trial design
- The study uses nurse for shifting/sharing some of the patient management tasks from physicians.
- The Intervention provides clinical decision support to physician for standardised and integrated management of hypertension and diabetes along with co-morbid conditions such as depression and alcohol use disorder.
- The mWellcare intervention is being implemented in the real world scenario aimed at utilising the existing human resource with an aim of scalability.

Limitations:

- Effectiveness of the trial will also depend on the availability of drugs recommended by the decision support system. Drugs are supplied by the state government and there are variations in availability across the state and districts, although such variations are likely to affect both arms equally.

For peer review only

Trial registration:

mWellcare trial is registered with Clinicaltrial.gov (Registration number NCT02480062) and Clinical Trial Registry of India (Registration number CTRI/2016/02/006641). The current version of the protocol is Version 2 dated 19th October 2015 and the study sponsor is Public Health Foundation of India, Gurgaon, India (www.phfi.org).

For peer review only

BACKGROUND AND RATIONALE

Cardiovascular disease (CVD) and diabetes are the leading causes of premature (<60 years) adult deaths in India with projections indicating almost 3-fold increase to 18 million premature years of life lost by 2030, a greater loss of life than the combined projected burdens for China, Russia and the USA.[1,2] CVD and diabetes will result in $2.32 trillion loss in national income in India between 2012 and 2030.[3] Primary care is considered as the appropriate setting for the prevention and control of chronic conditions and it is being strengthened through the National Program for Prevention and Control of cancer, diabetes, cardiovascular diseases and stroke (NPCDCS) in India.[2,4,5]

The telecom industry in India has grown exponentially over past fifteen years, from under 3.6 million mobile phone subscribers in March 2001 to over a billion subscribers in December 2015.[6] Mobile phones and other mobile technologies require less infrastructure than other eHealth systems, making them a promising investment for developing countries wanting to strengthen and transform their weak health systems, and to overcome health care worker shortages.[7–9] Systematic reviews have demonstrated a lack of robust evaluations of the impact of mHealth.[10–12] This lack of adequate evaluations led to need to provide robust evidence on the safety, benefits and associated cost-effectiveness of mHealth systems.[8,9,13] Systematic review and meta-analysis of studies evaluating role of decision support system for prevention of cardiovascular diseases show paucity of well-designed studies on patient outcomes.[14] In addition, there is a dearth of evidence on effect of task shifting/sharing strategies for CVD risk reduction and chronic disease management.[15,16] Through mWellcare we propose to enhance the quality of hypertension and diabetes care at primary care level in India by using mobile phone technology that implements a clinical decision support system and electronic health records for the use of healthcare professionals. While, the effectiveness of the mHealth system will be tested on a range of clinical and process-of-care indicators, the healthcare professionals in the control arm will receive “refresher” training in the management and follow up of hypertension and diabetes using the guidelines and charts.

METHODS AND ANALYSIS

Objective

The primary objective is to evaluate mWellcare system in two states in India over a twelve months period to determine its effectiveness on the management of patients with hypertension and/or diabetes based on their clinical outcomes (systolic blood pressure and HbA1c) compared to an enhanced care arm using a cluster randomized study design.

Secondary objective is to assess the impact of the mWellcare system on change in cardiovascular risk factors (BMI, Alcohol use, Tobacco use) and 10 year CVD risk score, mental health status, process outcomes and cost effectiveness.

Trial design

mWellcare is a cluster randomised controlled trial with equal allocation of participants between intervention and enhanced care arms.

Study setting

Twenty clusters each from two states – one in the north and the other in the south India have been selected for the trial. Each cluster is a Community Health Centre (CHC) which caters to a rural population of 120,000 and serves as a referral centre for four Primary Health Centres (PHCs) in the

For peer review only - http://bmjopen.bmj.com/site/about/guidelines.xhtml

formal healthcare delivery system.[17] CHCs were selected from districts covered under the National Program for the prevention and control of Cancer, Diabetes, Cardiovascular diseases and Stroke (NPCDCS). Allocation of the CHCs into intervention and control arm are described in the section on randomisation.

Inclusion criteria

Participants aged 30 years and above intending to reside in the catchment area of CHCs for at least next 12 months are eligible for the trial. Participants are included if they are diagnosed case of hypertension with blood pressure measuring ≥140/90mmHg OR type 2 diabetes mellitus (T2DM) with fasting blood sugar (FBS) ≥140mg/dL or post-prandial blood sugar (PPBS)≥200mg/dL and if they provide informed consent.

Exclusion criteria

Pregnant women, patients with type 1 diabetes, patients requiring immediate referral to tertiary care due to accelerated hypertension or diabetic complications, patients with learning difficulties or vision and/or hearing impairments, patient suffering from malignancy or life threatening disease with death probable in 4 years, patients not residing in the catchment area of the CHC.

Intervention

In the intervention arm, treatment and follow-up of recruited participants will be done using mWellcare system by the nurses and physicians. mWellcare system is a tablet computer based android application that is designed to store the health records electronically, provides decision-support recommendation tailored to the participant's compliance and risk level, enables long term monitoring and follow up and sends SMS reminders (to take medication and follow up visits) to patient. Development of intervention involved adapting existing clinical management guidelines to the local context, development and validation of clinical algorithm and pilot testing of mWellcare system. Nurses and physicians in the intervention arm were trained in the use of mWellcare system and also provided "refresher" training on the clinical management guidelines for hypertension and diabetes.

In the intervention arm, besides baseline information, the nurse enters information on co-morbid conditions – depression (using patient health questionnaire [PHQ-9]), alcohol use disorder (using alcohol use disorder identification test - AUDIT) and previous medication details into mWellcare system and generates & prints Decision Support Recommendation (DSR) for the physician. The DSR print out consist of patient profile, diagnosed condition, co-morbid conditions, previous and current medication and recommended treatment plan. It also provides lifestyle change recommendation and the date of the next follow up visit. After review of the DSR the physician may agree or suggest changes in the treatment plan which is recorded in the application. Nurse provides lifestyle advice brochure (in local language) and explains the same to each participant. Post recruitment, participants will be followed up for 12 months. Participants will receive SMS reminders for follow up visits scheduled as per clinical algorithm. During follow up visits, nurse will record relevant parameter to the patient's clinical record and generate DSR printout for physician's review. Each intervention site will get a monthly reports on number of participants reporting for scheduled follow up and average change in clinical parameters.

In the enhanced care arm, nurse and physicians were provided "refresher" training on the clinical management guidelines for hypertension and diabetes. In addition, charts on management of these

For peer review only - http://bmjopen.bmj.com/site/about/guidelines.xhtml

conditions were provided to the facilities for prominent display at the out-patient department. Physicians in the enhanced care arm, provides the management plan based on their assessment of clinical parameters of the participants. Nurse provides lifestyle advice brochure (in local language) and explain the same to each participant. Follow up in the enhanced care arm will be based on assessment of clinical parameters of the participants by the physician.

Outcomes

Primary outcomes:

- Difference in mean change (from baseline to one year) in systolic blood pressure between the two treatment arms.
- Difference in mean change (from baseline to one year) glycated haemoglobin (HbA1c) between the two treatment arms.

Secondary outcomes include:

- Difference in mean change (from baseline to one year) between the two treatment arms for fasting plasma glucose (FPG), total cholesterol and predicted 10 year risk of Cardiovascular disease (CVD) using recalibrated Framingham risk score
- Differences in risk factors such as: depression/anxiety; smoking behaviour; Body Mass Index (BMI); and Alcohol use between the two treatment arms.
- Comparison of costs associated with delivering the mWellcare intervention arm with respect to enhanced care.

Participant timeline

Enrolment and baseline assessment will be done during the first visit and the participants will be asked to come back for blood sample collection (for HbA1C & total cholesterol tests) within two weeks. The intervention will start on the first visit and continue for 12 months. After 12 months, endline assessment will be done.

Sample size calculation

The primary outcomes will be analysed as the average change of the biomarker (Systolic Blood Pressure (SBP) and Glycated Haemoglobin (HbA1c)) from baseline to 12 month follow up in each participant. In each of the trial arms there will be an average change and the effect of the intervention will be the difference of these averages.

A recruitment of 40 participants with hypertension per cluster in 40 clusters will yield over 98% power with a type I error of 5% to detect a mean difference of the change of 4 mmHg in SBP, between the intervention and enhanced care arm assuming a 15mmHg Standard Deviation (SD) of the changes in both arms and an Intra-class correlation (ICC)=0.05.[18] A number of 40 participants with diabetes per cluster will yield a power above 99% for detecting a true difference in average change of HbA1c between the two arms of 0.37%, assuming a 1.1% SD of the changes in both arms and an ICC=0.04.[19,20]

All calculations are based on the formula for comparison of two means in cluster-randomized trials proposed by Hayes and Bennett and assume a 20% loss to follow up.[21]

Recruitment

For the two primary outcomes (SBP and HbA1c), a total of 90 participants to be recruited per site with a minimum of 45 participants with each conditions – hypertension and diabetes. We estimated that the recruitment could be completed in 2 months enrolling a minimum of 2-3 participants a day.

For peer review only - http://bmjopen.bmj.com/site/about/guidelines.xhtml

However some sites had lower recruitment rate for either of the two conditions. Therefore, the final strategy was to stop recruitment of participants with either condition after reaching 45, then complete recruitment of patient with the other condition.

All individuals diagnosed to have hypertension and type 2 diabetes were assessed for eligibility by the nurse. All eligible participants were invited to participate in the trial. After providing written and verbal information about the trial, written consent was obtained by the nurse. The flow of trial participant is depicted in figure1.

Randomization

Randomization units are clusters (CHCs). The randomization list was generated by a statistician independent of the trial using STATA SE Version 12. The list was stratified by states (Haryana & Karnataka) and within states by availability/non-availability of nurses recruited under NPCDCS. In Karnataka, stratification based on availability of nurses was done allocating 10 clusters in each category (nurse available under NPCDCS/Not available). In Haryana, as there was no nurses recruited under NPCDCS, further stratification was not done. Clusters (CHCs) within each strata were randomised to intervention or enhanced care arm using block randomisation (with a block size of 2). Being a cluster randomized trial, only limited blinding could be enforced. The study statistician will remain blinded throughout the study until database is locked and study un-blinded.

Data collection, management and analysis

Baseline assessment

After consent, the nurse in both arms collected baseline data for the following parameters using a tablet computer: age, gender, marital status, occupation, alcohol use, smoking behaviour, health related quality of life measured using EuroQol five dimensions questionnaire (EQ-5D), height, weight, blood pressure and blood sugar. Consented participants were asked to return on a scheduled day for blood sample collection for HbA1c and total cholesterol.

Endline Assessment

Endline assessment of outcomes will be undertaken after completion of 12 month follow up. The assessments will be carried out by independent outcome assessors inviting the participants to the CHCs and mop up will be organised either through camps or home visits.

Data Management

This study will have only electronic version of the study forms and the dataset will automatically synchronise with the server. There will be built-in data checks and data cleaning will be done by trained data managers at PHFI, under the supervision of the Principal Investigator. All electronic data will be encrypted, password protected and stored in secure computer networks.

Data will be monitored centrally for its quality and completeness using electronic validation and on-site monitoring. Recruitment and retention of participants will be assessed by examining the number eligible for recruitment, number enrolled in the study and dropout from regular follow up at the completion of 12 months. In addition, we will carry out central statistical monitoring (CSM) all through the trial. This will aim to detect outliers and inliers, deviation of any cluster from the average and to detect potential data error or incorrect data collection procedure. Field coordinators will undertake monthly visits to all sites, record their observations using Objective Systematic Structured Observations (OSSO) checklist regarding intervention delivery, source documents

For peer review only - http://bmjopen.bmj.com/site/about/guidelines.xhtml

examination, protocol adherence and adverse event recording & reporting. The report will be sent online to the research coordinating centre. In addition, study investigators will undertake site visits to monitor enrolment process, intervention delivery and protocol adherence and report to the trial management committee.

Statistical Methods

Quantitative data analysis will be performed using STATA 13.0 (Statacorp Texas). A Statistical data analysis plan will be prepared and discussed with Data Safety Monitoring Board (DSMB) before data collection is completed.

The primary analyses will be conducted under the principle of intention to treat. All randomized participants will be analysed in the groups to which they were originally allocated to, regardless of whether they actually retained that specific group membership over the course of the trial or not. Participants who withdrew consent for use of their data will not be included in any analyses.

Demographic and clinical characteristics will be examined for participants at each CHC. Summaries will be presented as means and standard deviations of those variables that are approximately normally distributed, or medians and inter-quartiles for skewed variables. Categorical variables will be summarized as frequencies and percentages. Transformations will be used when distributional assumptions are not fulfilled for inferential tests on a continuous measure. If we find a considerable imbalance in some variables at baseline, we will consider adjusting for these variables by including them in the model to estimate the effect of the intervention. In principle, in a trial with individual blinded randomisation there should be no major baseline differences between arms, but this may not be the case in cluster sample given that the number of samples are small, individuals are not completely blind. So there is some potential for baseline differences between individuals recruited in each arm and this will have to be adjusted for in the analysis.

We will also account for the influence of clustering at the CHC level on the outcome. Data will be analysed at the individual level and comparisons will be made between the mean changes in the primary outcomes in the intervention and control groups. Linear mixed effect models adjusting for baseline measurements will be used with a random intercept for the CHC level to account for the clustering in the data.

Reporting of results will be in accordance with the principles of CONSORT statements extended for Cluster RCT.

Process evaluation

REAIM (reach, effectiveness, adoption, implementation and maintenance) framework for the process evaluation will be used for mWellcare trial.[22] This would entail a combination of qualitative (observations, interviews, focus group discussions) and quantitative (open ended and close ended questionnaires) assessment.

Fidelity will be assessed through logs of training, indicators from OSSO (Objective Systematic Structured Observation) checklist, and data captured through mWellcare system. Besides reports of monitoring visits by experts will form a part of the assessment.

Economic evaluation

To help inform potential financing and adoption of the mWellcare intervention, compared to enhanced care (control), we will estimate the value for different stakeholders: patients [mean

For peer review only - http://bmjopen.bmj.com/site/about/guidelines.xhtml

annual health expenditures in each arm]; healthcare providers [incremental costs to deliver the intervention and cost-effectiveness: e.g., incremental cost to prevent one CVD event]; and society [cost-utility: e.g., cost per quality-adjusted life years gained].

We will collect patient-level health care utilization and medical costs data incurred during the trial regarding outpatient visits, diagnostic services, medications, hospitalizations and lost productivity.[23] We will identify intervention costs from the NCD clinic and study expenditures (e.g., costs related to accessing the mWellcare system, and sending the regular SMS updates, training costs, labour costs for nurses, physician's time cost, and overhead costs minus costs attributable only to research activities [e.g., annual study visit]). We will compute the incremental costs of intervention (implementation, adverse-effects, medical care associated) compared to control. For effectiveness measures, we will use between-group differences in SBP and HbA1c reductions. Utility data will be collected using the EQ5D-VAS (EQ5D Visual Analogue Scale).

If the primary clinical outcomes (SBP and HbA1c) are shown to differ substantially, a full economic evaluation of the lifetime costs, benefits and cost effectiveness (in life years gained and quality adjusted life years gained) of switching from usual care to mWellcare intervention, using decision models will be performed.[24] Uncertainty around incremental costs effectiveness ratio (ICERs) will be estimated using non-parametric bootstrap methods. To inform societal perspective, we will calculate an incremental cost-utility ratio ($costs_{intervention} – costs_{control}$ / $utility_{intervention} – utility_{control}$).[25] To standardize for the time value of money, we will use a 3% annual discount rate. Costs will be expressed in Rupees and US$ (2016 value). We will conduct sensitivity analyses where discount rates, intervention costs, effectiveness, and other model parameters will be varied to estimate cost-utility under different scenarios.[26] We will also generate cost-effectiveness acceptability curve to demonstrate the probability of cost-effectiveness of the intervention at a range of willingness to pay threshold values. If no significant difference is found for the primary clinical outcomes a cost minimization analysis will be performed.

Data monitoring

A four member Data Safety Monitoring Board (DSMB) has been established. DSMB members are independent from sponsor and have no conflict of interest. The board members include experts in biostatistics, ethics and cardiovascular disease management. Based on observed beneficial or observed effect, DSMB will make recommendation to the Research Steering Group (RSG) in relation to the conduct of the trial (continue, change or terminate). The RSG takes responsibility for the design, conduct and analysis of the clinical trial. RSG is a multidisciplinary group who, collectively, has the scientific, medical and clinical trial management experience to conduct and evaluate the trial. In addition, a Research Management Committee (RMC) consisting of principal investigators, co-investigators, project manager and other team members have been formed to monitor progress of the study.

Serious Adverse Events (SAEs)

Occurrence of Serious Adverse Events (SAEs) that include severe hypoglycaemia, CVD events, diabetes related gangrene or amputation, end-stage renal disease and death will be monitored. Information about the occurrence of any adverse event will be sought at all scheduled visits, and outside of scheduled visits by participant self-report and tracking of non-study related visits. All

For peer review only - http://bmjopen.bmj.com/site/about/guidelines.xhtml

events reported in the study will be duly notified to the overviewing Ethics Committees and Data Safety Monitoring Board in the annual progress report.

Discussion

mWellcare is the first study in India, assessing the effectiveness of mHealth technology for integrated management of hypertension and diabetes at primary care level in a public health setting. Few trials have evaluated the impact of DSS on managing patients with hypertension and diabetes. A recent cluster randomized trial in Andhra Pradesh, India that evaluated the use of DSS in hypertension management, demonstrated significant reduction in systolic blood pressure among patient in primary health care facilities.[18] However, this trial focused only on hypertension while the other cardio-metabolic risk factors were not covered. The recently published results of CARRS Diabetes Trial shows significant improvement in diabetes care targets and cardio-metabolic risk reduction among patients who underwent multi-component quality improvement intervention comprising non-physician care coordinators and decision-support electronic health records.[27] However the findings of CARRS trial is confined to urban specialist care settings.

mWellcare trial with its cluster randomised design will extend the evidence on impact of mHealth intervention on patient's CVD outcomes at primary care level. It will provide evidence on the use of nurse for patient assessment and long term follow up using the mWellcare system thereby shifting/sharing some of the patient management tasks from physicians. It is also designed to provide decision support to physician for standardised and integrated management of hypertension and diabetes along with co-morbid condition such as depression and alcohol use disorder.

The mWellcare intervention is being implemented in the real world scenario aimed at utilising the existing human resource (as the nurse appointed at the out-patient clinics through the NPCDCS). The trial also incorporates cost-effectiveness analysis of the intervention. All these factors would be helpful in informing decision makers in allocating resources and scaling up the intervention based on the trial findings.

There are a few limitations for this study. The effectiveness of the trial will also depend on the availability of drugs, recommended by the decision support system, at the CHCs. Drugs are supplied by the state government and there are variations in availability across the state and districts, although such variations are likely to affect both arms equally. Another limitations is that the mWellcare system will be used only for the people who have been already diagnosed with hypertension or diabetes by the physician, and will not aid in opportunistic screening of patients coming to the CHC. This was done to keep both intervention and control arms at par. In case the intervention is scaled up, a screening module can be added to the mWellcare system.

Conclusion

The mWellcare trial will provide evidence on effectiveness of the nurse based mHealth intervention for integrated management of hypertension and diabetes at primary care level in India. Results from the trial will have direct policy relevance in adopting mHealth solution for managing CVDs at primary care level in India.

ETHICS AND DISSEMINATION

mWellcare trial protocol and study documents have been approved by the institutional Ethics Committee at Public Health Foundation of India (PHFI) and London School of Hygiene and Tropical

For peer review only - http://bmjopen.bmj.com/site/about/guidelines.xhtml

Medicine (LSHTM). All information collected as part of this study will be kept strictly confidential. Personal identifiers will be removed before transferring data for analysis. Participant's identity will be anonymous and post analysis of the data, the tapes and transcripts will be destroyed. Participant's name or identity will not be revealed in any of the publications arising from this research.

At the end of the study, the investigators will form a Publication, Presentation and Ancillary Studies (PP&A) subcommittee, which will develop a suitable policy protecting the rights of involved organisations regarding ownership of study materials and data. The full editorial control will reside with the Publication, Presentation and Ancillary Studies (PP&A) committee.

Competing interest

There is no conflict of interest between the funder and the authors.

Contributors

Dilip Jha – Coordinating the study, developed the first draft and subsequent revision of manuscript

Priti Gupta – Technical coordination of the study and helped in development & revision of manuscript

Ajay Vamadevan – Designed the study, providing overall supervision, reviewed of manuscript

Devraj Jindal – Contributed to development of mHealth solution and reviewed manuscript

Pablo Perel – Contributed to development of intervention and reviewed manuscript

David Prieto-Merino – Developed statistical analysis plan and reviewed manuscript

Jonathan Nyong – Contributed to development of cost effectiveness analysis plan, process indicators and reviewed manuscript

Vidya Venugopal – Contributed to development of statistical analysis plan and reviewed manuscript

Pramod Jacob - Contributed to development of information technology component of intervention and reviewed manuscript

Kavita Singh - Developed of cost effectiveness analysis plan and reviewed manuscript

Shifalika Goenka – Developed process indicators & analysis plan and reviewed manuscript

Ambuj Roy - Contributed to development of intervention and reviewed manuscript

Nikhil Tandon - Contributed to the design of the study, development of intervention and reviewed manuscript

Vikram Patel - Contributed to the design of the study, development of intervention and reviewed manuscript

Dorairaj Prabhakaran – Provided overall supervision, contributed to the design of the study, development of intervention and finalization of manuscript

Funding

This work was supported by the Wellcome Trust (Grant Number 096735/A/11/Z). The funding source had no role in the design of this study and will not have any role during its execution, analyses, interpretation of the data, or decision to submit results. However, if the mWellcare intervention is found to be effective in improving patient outcomes, the involving institutions – LSHTM, Wellcome Trust and PHFI – will commercialize the mWellcare system in a sustainable business model.

Data sharing statement

For peer review only - http://bmjopen.bmj.com/site/about/guidelines.xhtml

For peer review only

At the end of the study, a Publication, Presentation and Ancillary Studies (PP&A) subcommittee will be constituted which will develop a suitable policy for data sharing as per the funding agreement with The Wellcome Trust.

References

1 Lopez AD, Mathers CD, Ezzati M, *et al. Global Burden of Disease and Risk Factors*. The International Bank for Reconstruction and Development / The World Bank 2006.

2 Patel V, Chatterji S, Chisholm D, *et al.* Chronic diseases and injuries in India. *Lancet* 2011;**377**:413–28.

3 Bloom, D.E., Cafiero-Fonseca E.T., Candeias V AE, Bloom L., Gurfein L., Jané-Llopis E., Lubet, A., Mitgang E COJ, A S. Economics of Non-Communicable Diseases in India: The Costs and Returns on Investment of Interventions to Promote Healthy Living and Prevent, Treat, and Manage NCDs. 2014.

4 Beaglehole R, Epping-Jordan J, Patel V, *et al.* Improving the prevention and management of chronic disease in low-income and middle-income countries: a priority for primary health care. *Lancet* 2008;**372**:940–9.

5 National Program for Prevention and Control of cancer, diabetes, cardiovascular diseases and stroke (NPCDCS).

6 Telecom Regulatory Authority of India. The Indian Telecom Services Performance Indicators October - December, 2015. New Delhi: 2016.

7 Curioso WH. New Technologies and Public Health in Developing Countries: The Cell PREVEN Project. In: *The Internet and Health Care: Theory, Research and Practice*. Mahwah, NJ: Lawrence Erlbaum Associates 2006.

8 Curioso WH, Mechael PN. Enhancing 'M-Health' with South-South Collaborations. *Health Aff* 2010;**29**:264–7.

9 Vital Wave Consulting. mHealth for Development: The Opportunity of Mobile Technology for Healthcare in the Developing World. Washington DC and Berkshire, UK: 2009.

10 Blaya JA, Fraser HSF, Holt B. E-health technologies show promise in developing countries. *Health Aff* 2010;**29**:244–51.

11 Krishna S, Boren SA, Balas EA. Healthcare via cell phones: a systematic review. *Telemed J E Health* 2009;**15**:231–40.

12 Kaplan WA. Can the ubiquitous power of mobile phones be used to improve health outcomes in developing countries? *Global Health* 2006;**2**:9.

13 Rockfeller Foundation. Making the eHealth connection. 2008.

14 Anchala R, Pinto MP, Shroufi A, *et al.* The Role of Decision Support System (DSS) in Prevention of Cardiovascular Disease: A Systematic Review and Meta-Analysis. *PLoS One* 2012;**7**:e47064–e47064.

15 Ogedegbe G, Gyamfi J, Plange-Rhule J, *et al.* Task shifting interventions for cardiovascular risk reduction in low-income and middle-income countries: a systematic review of randomised controlled trials. *BMJ Open* 2014;**4**:e005983.

16 Joshi R, Alim M, Kengne AP, *et al.* Task shifting for non-communicable disease management in low and middle income countries--a systematic review. *PLoS One* 2014;**9**:e103754.

For peer review only - http://bmjopen.bmj.com/site/about/guidelines.xhtml

For peer review only

17 Ministry of Health & Family Welfare G of I. Rural Health Statistics 2015. 2015.

18 Anchala R, Kaptoge S, Pant H, *et al.* Evaluation of effectiveness and cost-effectiveness of a clinical decision support system in managing hypertension in resource constrained primary health care settings: results from a cluster randomized trial. *J Am Heart Assoc* 2015;**4**:e001213.

19 Tricco AC, Ivers NM, Grimshaw JM, *et al.* Effectiveness of quality improvement strategies on the management of diabetes: a systematic review and meta-analysis. *Lancet* 2012;**379**:2252–61.

20 Nair M, Ali MK, Ajay VS, *et al.* CARRS Surveillance study: design and methods to assess burdens from multiple perspectives. *BMC Public Health* 2012;**12**:701.

21 Hayes RJ, Bennett S. Simple sample size calculation for cluster-randomized trials. *Int J Epidemiol* 1999;**28**:319–26.

22 Glasgow R, Vogt T, Boles S. Evaluating the public health impact of health promotion interventions: the RE-AIM framework. *Am J Public Health* 1999;**89**:1322–7.

23 Drummond MF, Sculpher MJ, Torrance GW, *et al. Methods for the economic evaluation of health care programmes*. 2005.

24 Gilks WR, Richardson S, Spiegelhalter DJ. Markov Chain Monte Carlo in Practice. Technometrics. 1996;**39**:512.

25 Stone PW, Chapman RH, Sandberg EA, *et al.* Measuring costs in cost-utility analyses. Variations in the literature. *Int J Technol Assess Health Care* 2000;**16**:111–24.

26 Shillcutt SD, Walker DG, Goodman CA, *et al.* Cost effectiveness in low- and middle-income countries: a review of the debates surrounding decision rules. *Pharmacoeconomics* 2009;**27**:903–17.

27 Ali MK, Singh K, Kondal D, *et al.* Effectiveness of a Multicomponent Quality Improvement Strategy to Improve Achievement of Diabetes Care Goals: A Randomized, Controlled Trial. *Ann Intern Med* 2016;**165**:399–408.

For peer review only

For peer review only - http://bmjopen.bmj.com/site/about/guidelines.xhtml

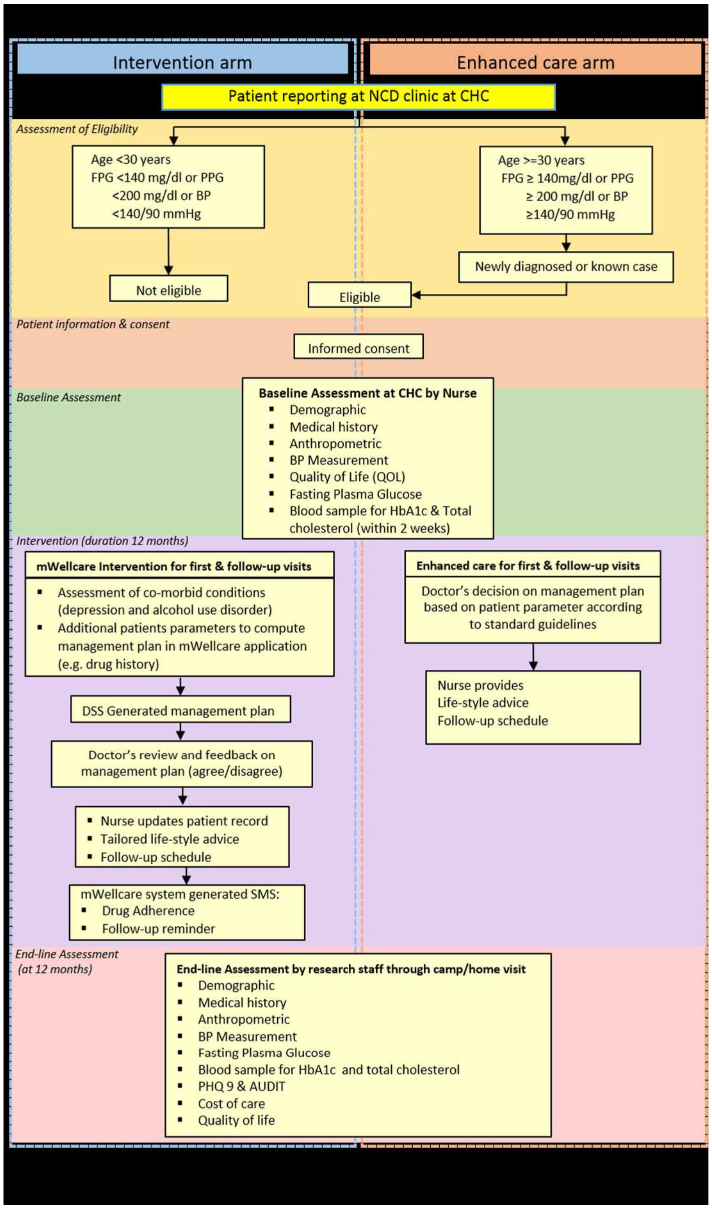

Trial Flowchart

159x270mm (300 x 300 DPI)

SPIRIT 2013 Checklist for mWellcare Trial Protocol

Section	Item No.	Description	Page No. / location in the manuscript
**Administrative Information**			
Title	1	Descriptive title identifying the study design, population, interventions, and, if applicable, trial acronym.	1
Trial Registration	2	Trial identifier and registry name. If not yet registered, name of intended registry	4
Protocol version	3	Date and version identifier	4
Funding	4	Sources and types of financial, material, and other support	12
Roles and responsibilities	5a	Names, affiliations, and roles of protocol contributors	12
	5b	Name and contact information for the trial sponsor	4
	5c	Role of study sponsor and funders, if any, in study design; collection, management, analysis, and interpretation of data; writing of the report; and the decision to submit the report for publication, including whether they will have ultimate authority over any of these activities.	12
	5d	Composition, roles, and responsibilities of the coordinating centre, steering committee, endpoint adjudication committee, data management team, and other individuals or groups overseeing the trial, if applicable (see Item 21a for Data Monitoring Committee).	10
**Introduction**			
Background and rationale	6a	Description of research question and justification for undertaking the trial, including summary of relevant studies (published and unpublished) examining benefits and harms for each intervention.	5
	6b	Explanation for choice of comparators.	5
Objectives	7	Specific objectives or hypotheses	5
Trial design	8	Description of trial design including type of trial (e.g., parallel group, crossover, factorial, single group), allocation ratio, and framework (e.g., superiority, equivalence, non-inferiority, exploratory)	5
**Methods: Participants, interventions, and outcomes**			
Study setting	9	Description of study settings (e.g., community clinic, academic hospital) and list of countries where data will be collected. Reference to where list of study sites can be obtained	5
Eligibility criteria	10	Inclusion and exclusion criteria for participants. If applicable, eligibility criteria for study centres and individuals who will perform the interventions (e.g., surgeons, psychotherapists).	6
Interventions	11a	Item 11a: Interventions for each group with sufficient detail to allow replication, including how and when they will be administered.	6

For peer review only

Section	Item No.	Description	Page No. / location in the manuscript
	11b	Criteria for discontinuing or modifying allocated interventions for a given trial participant (e.g., drug dose change in response to harms, participant request, or improving/worsening disease).	NA
	11c	Strategies to improve adherence to intervention protocols, and any procedures for monitoring adherence (e.g., drug tablet return; laboratory tests).	10
	11d	Relevant concomitant care and interventions that are permitted or prohibited during the trial.	NA
Outcomes	12	Primary, secondary, and other outcomes, including the specific measurement variable (e.g., systolic blood pressure), analysis metric (e.g., change from baseline, final value, time to event), method of aggregation (e.g., median, proportion), and time point for each outcome. Explanation of the clinical relevance of chosen efficacy and harm outcomes is strongly recommended.	7
Participant timeline	13	Time schedule of enrolment, interventions (including any run-ins and washouts), assessments, and visits for participants. A schematic diagram is highly recommended	7
Sample size	14	Estimated number of participants needed to achieve study objectives and how it was determined, including clinical and statistical assumptions supporting any sample size calculations	7
Recruitment	15	Strategies for achieving adequate participant enrolment to reach target sample size	7
**Assignment of intervention**			
Allocation	16a	Method of generating the allocation sequence (e.g., computer-generated random numbers), and list of any factors for stratification. To reduce predictability of a random sequence, details of any planned restriction (e.g., blocking) should be provided in a separate document that is unavailable to those who enroll participants or assign interventions.	8
	16b	Mechanism of implementing the allocation sequence (e.g., central telephone; sequentially numbered, opaque, sealed envelopes), describing any steps to conceal the sequence until interventions are assigned.	
Blinding	17a	Who will be blinded after assignment to interventions (e.g., trial participants, care providers, outcome assessors, data analysts), and how	8
	17b	If blinded, circumstances under which unblinding is permissible, and procedure for revealing a participant's allocated intervention during the trial.	

For peer review only

Section	Item No.	Description	Page No. / location in the manuscript
**Data collection, management, and analysis**			
Data collection methods	18a	Plans for assessment and collection of outcome, baseline, and other trial data, including any related processes to promote data quality (e.g., duplicate measurements, training of assessors) and a description of study instruments (e.g., questionnaires, laboratory tests) along with their reliability and validity, if known. Reference to where data collection forms can be found, if not in the protocol	8
	18b	Retention: Plans to promote participant retention and complete follow-up, including list of any outcome data to be collected for participants who discontinue or deviate from intervention protocols	8
Data management	19	Plans for data entry, coding, security, and storage, including any related processes to promote data quality (e.g., double data entry; range checks for data values). Reference to where details of data management procedures can be found, if not in the protocol	8
Statistical Methods	20a	Outcomes: Statistical methods for analysing primary and secondary outcomes. Reference to where other details of the statistical analysis plan can be found, if not in the protocol.	9
	20b	Additional analyses: Methods for any additional analyses (e.g., subgroup and adjusted analyses).	NA
	20c	Analysis population and missing data: Definition of analysis population relating to protocol non-adherence (e.g., as randomised analysis), and any statistical methods to handle missing data (e.g., multiple imputation)	NA
**Data Monitoring**			
Data monitoring	21a	Formal committee: Composition of data monitoring committee (DMC); summary of its role and reporting structure; statement of whether it is independent from the sponsor and competing interests; and reference to where further details about its charter can be found, if not in the protocol. Alternatively, an explanation of why a DMC is not needed	10
	21b	Interim analysis: Description of any interim analyses and stopping guidelines, including who will have access to these interim results and make the final decision to terminate the trial.	10
Harms	22	Plans for collecting, assessing, reporting, and managing solicited and spontaneously reported adverse events and other unintended effects of trial interventions or trial	10

For peer review only

Section	Item No.	Description	Page No. / location in the manuscript
		conduct.	
Auditing	23	Frequency and procedures for auditing trial conduct, if any, and whether the process will be independent from investigators and the sponsor.	8 &9
**Ethics and dissemination**			
Research ethics approval	24	Plans for seeking research ethics committee/institutional review board (REC/IRB) approval.	12
Protocol Amendment	25	Plans for communicating important protocol modifications (e.g., changes to eligibility criteria, outcomes, analyses) to relevant parties (e.g., investigators, REC/IRBs, trial participants, trial registries, journals, regulators).	NA
Consent	26a	Who will obtain informed consent or assent from potential trial participants or authorised surrogates, and how (see Item 32).	8
	26b	Ancillary studies: Additional consent provisions for collection and use of participant data and biological specimens in ancillary studies, if applicable	NA
Confidentiality	27	How personal information about potential and enrolled participants will be collected, shared, and maintained in order to protect confidentiality before, during, and after the trial.	11 & 12
Declaration of interests	28	Financial and other competing interests for principal investigators for the overall trial and each study site.	12
Access to data	29	Statement of who will have access to the final trial dataset, and disclosure of contractual agreements that limit such access for investigators.	12
Ancillary and post-trial care	30	Provisions, if any, for ancillary and post-trial care, and for compensation to those who suffer harm from trial participation	NA
Dissemination policy	31a	Trial results: Plans for investigators and sponsor to communicate trial results to participants, healthcare professionals, the public, and other relevant groups (e.g., via publication, reporting in results databases, or other data sharing arrangements), including any publication restrictions.	12
	31b	Authorship : Authorship eligibility guidelines and any intended use of professional writers	12
	31c	Reproducible Research: Plans, if any, for granting public access to the full protocol, participant-level dataset, and statistical code.	12

For peer review only

BMJ Open

Protocol for the mWellcare Trial: A multi-centre, cluster randomized, 12-month, controlled trial to compare the effectiveness of mWellcare, an mHealth system for an integrated management of patients with hypertension and diabetes, versus enhanced-usual care in India

Journal:	*BMJ Open*
Manuscript ID	bmjopen-2016-014851.R1
Article Type:	Protocol
Date Submitted by the Author:	25-May-2017

Complete List of Authors:	Jha, Dilip; Public Health Foundation of India,<br>Gupta, Priti; Public Health Foundation of India<br>Vamadevan, Ajay; Public Health Foundation of India<br>Jindal, Devraj; Public Health Foundation of India<br>Perel, Pablo; LSHTM, EPH<br>Prieto-Merino, David; London School of Hygiene & Tropical Medicine<br>Jacob, Pramod; Public Health Foundation of India<br>Nyong, Jonathan; LSHTM, EPH<br>Venugopal, Vidya; University of Pittsburgh Graduate School of Public Health, Epidemiology<br>Singh, Kavita; All India Institute of Medical Sciences, Endocrinology & Metabolism<br>Goenka, Shifalika; Public Health Foundation of India<br>Roy, Ambuj; All India Institute of Medical Sciences, Cardiology<br>Tandon, Nikhil; All India Institute of Medical Sciences,<br>Patel, Vikram; London School of Hygiene and Tropical Medicine<br>Prabhakaran, Dorairaj; Centre for Chronic Disease Control, ; Public Health Foundation of India,

**Primary Subject Heading**:	Cardiovascular medicine
Secondary Subject Heading:	Diabetes and endocrinology, Evidence based practice, Public health
Keywords:	Hypertension < CARDIOLOGY, mHealth, Type 2 Diabetes Mellitus, Decision Support System, Task shifting, Integrated management of chronic conditions

SCHOLARONE™ Manuscripts

Title

Protocol for the mWellcare Trial: A multi-centre, cluster randomized, 12-month, controlled trial to compare the effectiveness of mWellcare, an mHealth system for an integrated management of patients with hypertension and diabetes, versus enhanced-usual care in India

Author:

Dilip Jha, Priti Gupta, Vamadevan S Ajay, Devraj Jindal, Pablo Perel, David Prieto-Merino, Pramod Jacob, Jonathan Nyong, Vidya Venugopal, Kavita Singh, Shifalika Goenka, Ambuj Roy, Nikhil Tandon, Vikram Patel, Dorairaj Prabhakaran

Corresponding author:

Name - Prof Dorairaj Prabhakaran

Postal Address - Public Health Foundation of India (PHFI)
4th Floor, Plot - 47, Sector – 44
Gurgaon – 122 002 (Haryana) India

Email – dprabhakaran@ccdcindia.org

Telephone: +91-124-4781400

Fax: +91-124-4781601

Full name, department, institution, city and country of all co-authors.

Dilip Jha
Senior Research Project Manager
Centre for Control of Chronic Conditions
Public Health Foundation of India, Gurgaon, India

Priti Gupta
Senior Research Associate
Centre for Control of Chronic Conditions
Public Health Foundation of India, Gurgaon, India

Vamadevan S Ajay
Senior Research Scientist
Centre for Control of Chronic Conditions
Public Health Foundation of India, Gurgaon, India

Devraj Jindal
Project Manager
Centre for Control of Chronic Conditions
Public Health Foundation of India, Gurgaon, India

Pablo Perel
Associate Professor
Centre for Control of Chronic Conditions
London School of Hygiene & Tropical Medicine, London, UK

David Prieto-Merino
Associate Professor,
Centre for Control of Chronic Conditions
London School of Hygiene & Tropical Medicine, London, UK

For peer review only - http://bmjopen.bmj.com/site/about/guidelines.xhtml

Pramod Jacob
Health Informatics Consultant
Centre for Control of Chronic Conditions
Public Health Foundation of India, Gurgaon, India

Jonathan Nyong,
Research Fellow
Centre for Control of Chronic Conditions
London School of Hygiene & Tropical Medicine, London, UK

Vidya Venugopal
Postdoctoral Associate
Department of Epidemiology
University of Pittsburgh Graduate School of Public Health, Pittsburgh, USA

Kavita Singh
PhD Student
Centre for Control of Chronic Conditions
All India Institute of Medical Science, New Delhi, India

Shifalika Goenka,
Additional Professor
Centre for Control of Chronic Conditions
Public Health Foundation of India, Gurgaon, India

Ambuj Roy
Additional Professor, Department of Cardiology
Centre for Control of Chronic Conditions
All India Institute of Medical Science, New Delhi, India

Nikhil Tandon
Professor& Head, Department of Endocrinology & Metabolism,
Centre for Control of Chronic Conditions
All India Institute of Medical Science, New Delhi, India

Vikram Patel,
Professor
Centre for Control of Chronic Conditions
London School of Hygiene & Tropical Medicine, London, UK

Dorairaj Prabhakaran
Professor
Centre for Control of Chronic Conditions
Public Health Foundation of India, Gurgaon, India

Word count excluding title page, abstract, references, figures and tables: 4918

Keywords
Hypertension, diabetes, mHealth, decision support system, integrated management of chronic conditions

For peer review only

Abstract

Introduction

Rising burden of cardiovascular disease and diabetes is a major challenge to the health system in India. Innovative approaches such as mobile phone technology (mHealth) for electronic decision support in delivering evidence based and integrated care for hypertension, diabetes and comorbid depression has potential to transform the primary healthcare system.

Methods and analysis

mWellcare trial is a multi-centre, cluster randomized controlled trial evaluating the clinical and cost effectiveness of a mHealth system and nurse managed care for people with hypertension and diabetes in rural India. mWellcare system is an android-based mobile application designed to generate algorithm based clinical management prompts for treating hypertension and diabetes and also capable of storing health records, sending alerts and reminders for follow-up and adherence to medication. We recruited a total of 3702 participants from 40 Community Health Centres (CHCs); with >=90 at each of the CHCs in the intervention and control (enhanced care) arms. The primary outcome is the difference in mean change (from baseline to one year) in systolic blood pressure and glycated haemoglobin (HbA1c) between the two treatment arms. The secondary outcomes are difference in mean change from baseline to one year in fasting plasma glucose (FPG), total cholesterol, predicted 10 year risk of Cardio-vascular disease (CVD), depression, smoking behavior, body mass index (BMI) and alcohol use between the two treatment arms and cost effectiveness.

Ethics and dissemination

The study has been approved by the institutional Ethics Committees at Public Health Foundation of India and the London School of Hygiene and Tropical Medicine. Findings will be disseminated widely through peer reviewed publications, conference presentations and other mechanisms.

Strength & limitations of the study

Strength:

- mWellcare trial is the first study in India, assessing the effectiveness of mHealth technology for integrated management of hypertension and diabetes at primary care level in a public health setting.
- The study follows a cluster randomised controlled trial design
- The study uses nurse for shifting/sharing some of the patient management tasks from physicians.
- The Intervention provides clinical decision support to physician for standardised and integrated management of hypertension and diabetes along with co-morbid conditions such as depression and alcohol use disorder.
- The mWellcare intervention is being implemented in the real world scenario aimed at utilising the existing human resource with an aim of scalability.

Limitations:

- Effectiveness of the trial will also depend on the availability of drugs recommended by the decision support system. Drugs are supplied by the state government and there are variations in availability across the state and districts, although such variations are likely to affect both arms equally.

For peer review only

Trial registration:

mWellcare trial is registered with Clinicaltrial.gov (Registration number NCT02480062) and Clinical Trial Registry of India (Registration number CTRI/2016/02/006641). The current version of the protocol is Version 2 dated 19th October 2015 and the study sponsor is Public Health Foundation of India, Gurgaon, India (www.phfi.org).

For peer review only

For peer review only - http://bmjopen.bmj.com/site/about/guidelines.xhtml

BACKGROUND AND RATIONALE

Cardiovascular disease (CVD) and diabetes are the leading causes of premature (<60 years) adult deaths in India with projections indicating almost 3-fold increase to 18 million premature years of life lost by 2030, a greater loss of life than the combined projected burdens for China, Russia and the USA.[1,2] CVD and diabetes will result in $2.32 trillion loss in national income in India between 2012 and 2030.[3] Primary care is considered as the appropriate setting for the prevention and control of chronic conditions and it is being strengthened through the National Program for Prevention and Control of cancer, diabetes, cardiovascular diseases and stroke (NPCDCS) in India.[2,4,5]

The telecom industry in India has grown exponentially over past fifteen years, from under 3.6 million mobile phone subscribers in March 2001 to over a billion subscribers in December 2015.[6] Mobile phones and other mobile technologies require less infrastructure than other eHealth systems, making them a promising investment for developing countries wanting to strengthen and transform their weak health systems, and to overcome health care worker shortages.[7–9] Systematic reviews have demonstrated a lack of robust evaluations of the impact of mHealth.[10–14] This lack of adequate evaluations led to need to provide robust evidence on the safety, benefits and associated cost-effectiveness of mHealth systems.[8,9,15,16] Systematic review and meta-analysis of studies evaluating role of decision support system for prevention of cardiovascular diseases show paucity of well-designed studies on patient outcomes.[17] In addition, there is a dearth of evidence on effect of task shifting/sharing strategies for CVD risk reduction and chronic disease management.[18,19] Through mWellcare we propose to enhance the quality of hypertension and diabetes care at primary care level in India by using mobile phone technology that implements a clinical decision support system and electronic health records for the use of healthcare professionals. The effectiveness of the mHealth system will be tested on a range of clinical and process-of-care indicators.

METHODS AND ANALYSIS

Objective

The primary objective is to evaluate mWellcare system in two states in India over a twelve months period to determine its effectiveness on the management of patients with hypertension and/or diabetes based on their clinical outcomes (systolic blood pressure and HbA1c) compared to an enhanced care arm using a cluster randomized study design.

Secondary objective is to assess the impact of the mWellcare system on change in cardiovascular risk factors (BMI, Alcohol use, Tobacco use) and 10 year CVD risk score, mental health status, process outcomes and cost effectiveness.

Trial design

mWellcare is a cluster randomised controlled trial with equal allocation of participants between intervention and enhanced care arms.

Study setting

Twenty clusters each from two states – one in the north and the other in the south India have been selected for the trial. Each cluster is a Community Health Centre (CHC) which caters to a rural population of 120,000 and serves as a referral centre for four Primary Health Centres (PHCs) in the formal healthcare delivery system.[20] CHCs were selected from districts covered under the National

For peer review only - http://bmjopen.bmj.com/site/about/guidelines.xhtml

Program for the prevention and control of Cancer, Diabetes, Cardiovascular diseases and Stroke (NPCDCS). Allocation of the CHCs into intervention and control arm are described in the section on randomisation.

Inclusion criteria

Participants aged 30 years and above intending to reside in the catchment area of CHCs for at least next 12 months were eligible for the trial. Participants were included if they were diagnosed case of hypertension with blood pressure measuring ≥140/90mmHg OR type 2 diabetes mellitus (T2DM) with fasting blood sugar (FBS) ≥140mg/dL or post-prandial blood sugar (PPBS)≥200mg/dL and if they provided informed consent.

Exclusion criteria

Pregnant women, patients with type 1 diabetes, patients requiring immediate referral to tertiary care due to accelerated hypertension or diabetic complications, patients with learning difficulties or vision and/or hearing impairments, patient suffering from malignancy or life threatening disease with death probable in 4 years, patients not residing in the catchment area of the CHC were excluded.

Intervention

In the intervention arm, nurse and physician are providing treatment and follow-up to recruited participants using mWellcare system. mWellcare system is a tablet computer based android application that is designed to store the health records electronically, provides decision-support recommendation tailored to the participant's compliance and risk level, enables long term monitoring and follow up and sends SMS reminders (to take medication and follow up visits) to patient. Development of intervention involved adapting existing clinical management guidelines to the local context, development and validation of clinical algorithm and pilot testing of mWellcare system. Nurses and physicians in the intervention arm were trained in the use of mWellcare system and also provided "refresher" training on the clinical management guidelines for hypertension and diabetes.

In the intervention arm, besides baseline information, the nurse enters information on co-morbid conditions – depression (using patient health questionnaire [PHQ-9]), alcohol use disorder (using alcohol use disorder identification test - AUDIT) and previous medication details into mWellcare system and generates & prints Decision Support Recommendation (DSR) for the physician. The DSR print-out consists of patient profile, diagnosed condition, co-morbid conditions, previous and current medication and recommended treatment plan. It also provides lifestyle change recommendation and the date of the next follow up visit. After review of the DSR the physician may agree or suggest changes in the treatment plan which is recorded in the application. Nurse provides lifestyle advice brochure (in local language) and explains the same to each participant. Each participants will be followed up for 12 months. During this period, participants will receive SMS reminders for follow up visits scheduled as per clinical algorithm. During follow up visits, nurse enters relevant parameter to the patient's clinical record and generates DSR printout for physician's review. Each intervention site gets a monthly reports on number of participants reporting for scheduled follow up and average change in clinical parameters.

In the enhanced care arm, nurse and physicians were provided "refresher" training on the clinical management guidelines for hypertension and diabetes. In addition, charts on management of these

For peer review only - http://bmjopen.bmj.com/site/about/guidelines.xhtml

conditions were provided to the facilities for prominent display at the out-patient department. Physicians in the enhanced care arm, provides the management plan based on their assessment of clinical parameters of the participants. Nurse provides lifestyle advice brochure (in local language) and explain the same to each participant. Follow up in the enhanced care arm is based on assessment of clinical parameters of the participants by the physician.

Outcomes

Primary outcomes:

- Difference in mean change (from baseline to one year) in systolic blood pressure between the two treatment arms.
- Difference in mean change (from baseline to one year) glycated haemoglobin (HbA1c) between the two treatment arms.

Secondary outcomes include:

- Difference in mean change (from baseline to one year) between the two treatment arms for fasting plasma glucose (FPG), total cholesterol and predicted 10 year risk of Cardiovascular disease (CVD) using recalibrated Framingham risk score
- Differences in risk factors such as: depression/anxiety; smoking behaviour; Body Mass Index (BMI); and Alcohol use between the two treatment arms.
- Comparison of costs associated with delivering the mWellcare intervention arm with respect to enhanced care.

Participant timeline

Enrolment and baseline assessment were done during the first visit and the participants were asked to come back for blood sample collection (for HbA1C & total cholesterol tests) within two weeks. The intervention started on the first visit and will continue for 12 months. After 12 months, endline assessment will be done.

Sample size calculation

The primary outcomes will be analysed as the average change of the biomarker (Systolic Blood Pressure (SBP) and Glycated Haemoglobin (HbA1c)) from baseline to 12 month follow up in each participant. In each of the trial arms there will be an average change and the effect of the intervention will be the difference of these averages.

A recruitment of 40 participants with hypertension per cluster in 40 clusters will yield over 98% power with a type I error of 5% to detect a mean difference of the change of 4 mmHg in SBP, between the intervention and enhanced care arm assuming a 15mmHg Standard Deviation (SD) of the changes in both arms and an Intra-class correlation (ICC)=0.05.[21] A number of 40 participants with diabetes per cluster will yield a power above 99% for detecting a true difference in average change of HbA1c between the two arms of 0.37%, assuming a 1.1% SD of the changes in both arms and an ICC=0.04.[22,23]

All calculations are based on the formula for comparison of two means in cluster-randomized trials proposed by Hayes and Bennett and assume a 20% loss to follow up.[24]

Recruitment

For the two primary outcomes (SBP and HbA1c), we planned to recruit a total of 90 participants per site with a minimum of 45 participants with each conditions – hypertension and diabetes. We estimated that the recruitment could be completed in 2 months enrolling a minimum of 2-3

For peer review only - http://bmjopen.bmj.com/site/about/guidelines.xhtml

participants a day. However some sites had lower recruitment rate for either of the two conditions. Therefore, the final strategy was to stop recruitment of participants with either condition after reaching 45, then complete recruitment of patient with the other condition. A total of 3702 participants were recruited from 40 CHCs.

All individuals diagnosed to have hypertension and/or type 2 diabetes were assessed for eligibility by the nurse. All eligible participants were invited to participate in the trial. After providing written and verbal information about the trial, written consent was obtained by the nurse. The flow of trial participant is depicted in figure1.

Randomization

Randomization units are clusters (CHCs). The randomization list was generated by a statistician independent of the trial using STATA SE Version 12. The list was stratified by states (Haryana & Karnataka) and within states by availability/non-availability of nurses recruited under NPCDCS. In Karnataka, stratification based on availability of nurses was done allocating 10 clusters in each category (nurse available under NPCDCS/Not available). In Haryana, as there was no nurses recruited under NPCDCS, further stratification was not done. Clusters (CHCs) within each strata were randomised to intervention or enhanced care arm using block randomisation (with a block size of 2). Being a cluster randomized trial, only limited blinding could be enforced. The study statistician will remain blinded throughout the study until database is locked and study un-blinded.

Data collection, management and analysis

Baseline assessment

After consent, the nurse in both arms collected baseline data for the following parameters using a tablet computer: age, gender, marital status, occupation, alcohol use, smoking behaviour, health related quality of life measured using EuroQol five dimensions questionnaire (EQ-5D), height, weight, blood pressure and blood sugar. Consented participants were asked to return on a scheduled day for blood sample collection for HbA1c and total cholesterol.

Endline Assessment

Endline assessment of outcomes will be undertaken after completion of 12 month follow up. The assessments will be carried out by independent outcome assessors inviting the participants to camps and mop up will be organised through home visits.

Data Management

This study has only electronic version of the study forms and the dataset automatically synchronises with the server. There are built-in data checks and further data cleaning will be done by trained data managers at PHFI, under the supervision of the Principal Investigator. All electronic data are encrypted, password protected and stored in secure computer networks.

Data is monitored centrally for its quality and completeness using electronic validation and on-site monitoring. Recruitment and retention of participants was assessed by examining the number eligible for recruitment, number enrolled in the study and dropout from regular follow up will be assessed at the completion of 12 months. In addition, we are carrying out central statistical monitoring (CSM) all through the trial. CSM aims to detect outliers and inliers, deviation of any cluster from the average and to detect potential data error or incorrect data collection procedure. Field coordinators undertake monthly visits to all sites, record their observations using Objective

For peer review only - http://bmjopen.bmj.com/site/about/guidelines.xhtml

Systematic Structured Observations (OSSO) checklist regarding intervention delivery, source documents examination, protocol adherence and adverse event recording & reporting. The monthly visit reports are sent online to the research coordinating centre. In addition, study investigators undertook site visits to monitor enrolment process, intervention delivery and protocol adherence and reported to the trial management committee.

Statistical Methods

Quantitative data analysis will be performed using STATA 13.0 (Statacorp Texas). A Statistical data analysis plan will be prepared and discussed with Data Safety Monitoring Board (DSMB) before data collection is completed.

The primary analyses will be conducted under the principle of intention to treat. All randomized participants will be analysed in the groups to which they were originally allocated to, regardless of whether they actually retained that specific group membership over the course of the trial or not. Participants who withdrew consent for use of their data will not be included in any analyses.

Demographic and clinical characteristics will be examined for participants at each CHC. Summaries will be presented as means and standard deviations of those variables that are approximately normally distributed, or medians and inter-quartiles for skewed variables. Categorical variables will be summarized as frequencies and percentages. Transformations will be used when distributional assumptions are not fulfilled for inferential tests on a continuous measure. If we find a considerable imbalance in some variables at baseline, we will consider adjusting for these variables by including them in the model to estimate the effect of the intervention. In principle, in a trial with individual blinded randomisation there should be no major baseline differences between arms, but this may not be the case in cluster sample given that the number of samples are small, individuals are not completely blind. So there is some potential for baseline differences between individuals recruited in each arm and this will have to be adjusted for in the analysis.

We will also account for the influence of clustering at the CHC level on the outcome. Data will be analysed at the individual level and comparisons will be made between the mean changes in the primary outcomes in the intervention and control groups. Linear mixed effect models adjusting for baseline measurements will be used with a random intercept for the CHC level to account for the clustering in the data.

Reporting of results will be in accordance with the principles of CONSORT statements extended for Cluster RCT.

Process evaluation

REAIM (reach, effectiveness, adoption, implementation and maintenance) framework for the process evaluation will be used for mWellcare trial.[25] This would entail a combination of qualitative (observations, interviews, focus group discussions) and quantitative (open ended and close ended questionnaires) assessment.

Fidelity will be assessed through logs of training, indicators from OSSO (Objective Systematic Structured Observation) checklist, and data captured through mWellcare system. Besides reports of monitoring visits by experts will form a part of the assessment.

Economic evaluation

For peer review only - http://bmjopen.bmj.com/site/about/guidelines.xhtml

To help inform potential financing and adoption of the mWellcare intervention, compared to enhanced care (control), we will estimate the value for different stakeholders: patients [mean annual health expenditures in each arm]; healthcare providers [incremental costs to deliver the intervention and cost-effectiveness: e.g., incremental cost to prevent one CVD event]; and society [cost-utility: e.g., cost per quality-adjusted life years gained].

We will collect patient-level health care utilization and medical costs data incurred during the trial regarding outpatient visits, diagnostic services, medications, hospitalizations and lost productivity.[26] We will identify intervention costs from the NCD clinic and study expenditures (e.g., costs related to accessing the mWellcare system, and sending the regular SMS updates, training costs, labour costs for nurses, physician's time cost, and overhead costs minus costs attributable only to research activities [e.g., annual study visit]). We will compute the incremental costs of intervention (implementation, adverse-effects, medical care associated) compared to control. For effectiveness measures, we will use between-group differences in SBP and HbA1c reductions. Utility data will be collected using the EQ5D-VAS (EQ5D Visual Analogue Scale).

If the primary clinical outcomes (SBP and HbA1c) are shown to differ substantially, a full economic evaluation of the lifetime costs, benefits and cost effectiveness (in life years gained and quality adjusted life years gained) of switching from usual care to mWellcare intervention, using decision models will be performed.[27] Uncertainty around incremental costs effectiveness ratio (ICERs) will be estimated using non-parametric bootstrap methods. To inform societal perspective, we will calculate an incremental cost-utility ratio ($costs_{intervention}$ – $costs_{control}$ / $utility_{intervention}$ – $utility_{control}$).[28] To standardize for the time value of money, we will use a 3% annual discount rate. Costs will be expressed in Rupees and US$ (2016 value). We will conduct sensitivity analyses where discount rates, intervention costs, effectiveness, and other model parameters will be varied to estimate cost-utility under different scenarios.[29] We will also generate cost-effectiveness acceptability curve to demonstrate the probability of cost-effectiveness of the intervention at a range of willingness to pay threshold values. If no significant difference is found for the primary clinical outcomes a cost minimization analysis will be performed.

Data monitoring

A four member Data Safety Monitoring Board (DSMB) has been established. DSMB members are independent from sponsor and have no conflict of interest. The board members include experts in biostatistics, ethics and cardiovascular disease management. Based on observed beneficial or observed effect, DSMB will make recommendation to the Research Steering Group (RSG) in relation to the conduct of the trial (continue, change or terminate). The RSG takes responsibility for the design, conduct and analysis of the clinical trial. RSG is a multidisciplinary group who, collectively, has the scientific, medical and clinical trial management experience to conduct and evaluate the trial. In addition, a Research Management Committee (RMC) consisting of principal investigators, co-investigators, project manager and other team members have been formed to monitor progress of the study.

Serious Adverse Events (SAEs)

Occurrence of Serious Adverse Events (SAEs) that include severe hypoglycaemia, CVD events, diabetes related gangrene or amputation, end-stage renal disease and death is being monitored. Information about the occurrence of any adverse event is sought at all scheduled visits, and outside

For peer review only - http://bmjopen.bmj.com/site/about/guidelines.xhtml

of scheduled visits by participant self-report and tracking of non-study related visits. All events reported in the study will be duly notified to the overviewing Ethics Committees and Data Safety Monitoring Board in the annual progress report.

Discussion

mWellcare is the first study in India, assessing the effectiveness of mHealth technology for integrated management of hypertension and diabetes at primary care level in a public health setting. Few trials have evaluated the impact of DSS on managing patients with hypertension and diabetes. A recent cluster randomized trial in Andhra Pradesh, India that evaluated the use of DSS in hypertension management, demonstrated significant reduction in systolic blood pressure among patient in primary health care facilities.[21] However, this trial focused only on hypertension while the other cardio-metabolic risk factors were not covered. The recently published results of CARRS Diabetes Trial shows significant improvement in diabetes care targets and cardio-metabolic risk reduction among patients who underwent multi-component quality improvement intervention comprising non-physician care coordinators and decision-support electronic health records.[30] However the findings of CARRS trial is confined to urban specialist care settings.

mWellcare trial with its cluster randomised design will extend the evidence on impact of mHealth intervention on patient's CVD outcomes at primary care level. It will provide evidence on the use of nurse for patient assessment and long term follow up using the mWellcare system thereby shifting/sharing some of the patient management tasks from physicians. It is also designed to provide decision support to physician for standardised and integrated management of hypertension and diabetes along with co-morbid condition such as depression and alcohol use disorder.

The mWellcare intervention is being implemented in the real world scenario aimed at utilising the existing human resource (as the nurse appointed at the out-patient clinics through the NPCDCS). The trial also incorporates cost-effectiveness analysis of the intervention. All these factors would be helpful in informing decision makers in allocating resources and scaling up the intervention based on the trial findings.

There are a few limitations for this study. The effectiveness of the trial will also depend on the availability of drugs, recommended by the decision support system, at the CHCs. Drugs are supplied by the state government and there are variations in availability across the state and districts, although such variations are likely to affect both arms equally. Another limitations is that the mWellcare system will be used only for the people who have been already diagnosed with hypertension or diabetes by the physician, and will not aid in opportunistic screening of patients coming to the CHC. This was done to keep both intervention and control arms at par. In case the intervention is scaled up, a screening module can be added to the mWellcare system.

Conclusion

The mWellcare trial will provide evidence on effectiveness of the nurse based mHealth intervention for integrated management of hypertension and diabetes at primary care level in India. Results from the trial will have direct policy relevance in adopting mHealth solution for managing CVDs at primary care level in India.

ETHICS AND DISSEMINATION

For peer review only - http://bmjopen.bmj.com/site/about/guidelines.xhtml

mWellcare trial protocol and study documents have been approved by the institutional Ethics Committee at Public Health Foundation of India (PHFI) and London School of Hygiene and Tropical Medicine (LSHTM). All information collected as part of this study will be kept strictly confidential. Personal identifiers will be removed before transferring data for analysis. Participant's identity will be anonymous and post analysis of the data, the tapes and transcripts will be destroyed. Participant's name or identity will not be revealed in any of the publications arising from this research.

Findings from this study will be submitted for publication in peer-reviewed journals. The study results will be shared with health professionals (primary care providers), local government, and decision makers as brief policy notes. The study investigators will also disseminate findings through professional conferences targeting primary and secondary care physicians, research community and public health policy-makers more widely. The results of this study will provide policy-relevant recommendations for the uptake of m-Health interventions in the management of hypertension and diabetes in India.

At the end of the study, the investigators will form a Publication, Presentation and Ancillary Studies (PP&A) subcommittee, which will develop a suitable policy protecting the rights of involved organisations regarding ownership of study materials and data. The full editorial control will reside with the Publication, Presentation and Ancillary Studies (PP&A) committee. All investigators will be given access to the cleaned data sets.

Competing interest

There is no conflict of interest between the funder and the authors.

Contributors

Dilip Jha – Coordinating the study, developed the first draft and subsequent revision of manuscript

Priti Gupta – Technical coordination of the study and helped in development & revision of manuscript

Ajay Vamadevan – Designed the study, providing overall supervision, reviewed of manuscript

Devraj Jindal – Contributed to development of mHealth solution and reviewed manuscript

Pablo Perel – Contributed to development of intervention and reviewed manuscript

David Prieto-Merino – Developed statistical analysis plan and reviewed manuscript

Jonathan Nyong – Contributed to development of cost effectiveness analysis plan, process indicators and reviewed manuscript

Vidya Venugopal – Contributed to development of statistical analysis plan and reviewed manuscript

Pramod Jacob - Contributed to development of information technology component of intervention and reviewed manuscript

Kavita Singh - Developed of cost effectiveness analysis plan and reviewed manuscript

Shifalika Goenka – Developed process indicators & analysis plan and reviewed manuscript

Ambuj Roy - Contributed to development of intervention and reviewed manuscript

Nikhil Tandon - Contributed to the design of the study, development of intervention and reviewed manuscript

Vikram Patel - Contributed to the design of the study, development of intervention and reviewed manuscript

For peer review only - http://bmjopen.bmj.com/site/about/guidelines.xhtml

For peer review only

Dorairaj Prabhakaran – Provided overall supervision, contributed to the design of the study, development of intervention and finalization of manuscript

Funding

This work was supported by the Wellcome Trust (Grant Number 096735/A/11/Z). The funding source had no role in the design of this study and will not have any role during its execution, analyses, interpretation of the data, or decision to submit results. However, if the mWellcare intervention is found to be effective in improving patient outcomes, the involving institutions – LSHTM, Wellcome Trust and PHFI – will commercialize the mWellcare system in a sustainable business model.

Data sharing statement

At the end of the study, a Publication, Presentation and Ancillary Studies (PP&A) subcommittee will be constituted which will develop a suitable policy for data sharing as per the funding agreement with The Wellcome Trust.

References

1 Lopez AD, Mathers CD, Ezzati M, *et al. Global Burden of Disease and Risk Factors*. The International Bank for Reconstruction and Development / The World Bank 2006.

2 Patel V, Chatterji S, Chisholm D, *et al.* Chronic diseases and injuries in India. *Lancet* 2011;**377**:413–28.

3 Bloom, D.E., Cafiero-Fonseca E.T., Candeias V AE, Bloom L., Gurfein L., Jané-Llopis E., Lubet, A., Mitgang E COJ, A S. Economics of Non-Communicable Diseases in India: The Costs and Returns on Investment of Interventions to Promote Healthy Living and Prevent, Treat, and Manage NCDs. 2014.

4 Beaglehole R, Epping-Jordan J, Patel V, *et al.* Improving the prevention and management of chronic disease in low-income and middle-income countries: a priority for primary health care. *Lancet* 2008;**372**:940–9.

5 National Program for Prevention and Control of cancer, diabetes, cardiovascular diseases and stroke (NPCDCS).

6 Telecom Regulatory Authority of India. The Indian Telecom Services Performance Indicators October - December, 2015. New Delhi: 2016.

7 Curioso WH. New Technologies and Public Health in Developing Countries: The Cell PREVEN Project. In: *The Internet and Health Care: Theory, Research and Practice*. Mahwah, NJ: Lawrence Erlbaum Associates 2006.

8 Curioso WH, Mechael PN. Enhancing 'M-Health' with South-South Collaborations. *Health Aff* 2010;**29**:264–7.

9 Vital Wave Consulting. mHealth for Development: The Opportunity of Mobile Technology for Healthcare in the Developing World. Washington DC and Berkshire, UK: 2009.

10 Blaya JA, Fraser HSF, Holt B. E-health technologies show promise in developing countries. *Health Aff* 2010;**29**:244–51.

11 Krishna S, Boren SA, Balas EA. Healthcare via cell phones: a systematic review. *Telemed J E Health* 2009;**15**:231–40.

For peer review only

12 Kaplan WA. Can the ubiquitous power of mobile phones be used to improve health outcomes in developing countries? *Global Health* 2006;**2**:9.

13 Peiris, D., Praveen, D., Johnson C et al. Use of mHealth Systems and Tools for Non-Communicable Diseases in Low- and Middle-Income Countries: a Systematic Review. *J Cardiovasc Trans Res* 2014;**7**:677–91.

14 Free C, Phillips G, Watson L, *et al.* The Effectiveness of Mobile-Health Technologies to Improve Health Care Service Delivery Processes: A Systematic Review and Meta-Analysis. *PLoS Med* 2013;**10**. doi:10.1371/journal.pmed.1001363

15 Rockfeller Foundation. Making the eHealth connection. 2008.

16 Hall AK, Cole-Lewis H BJ. Mobile Text Messaging for Health: A Systematic Review of Reviews. *Annu Rev Public Heal* 2015;**36**:393–415. doi:10.1146/annurev-publhealth-031914-122855.Mobile

17 Anchala R, Pinto MP, Shroufi A, *et al.* The Role of Decision Support System (DSS) in Prevention of Cardiovascular Disease: A Systematic Review and Meta-Analysis. *PLoS One* 2012;**7**:e47064–e47064.

18 Ogedegbe G, Gyamfi J, Plange-Rhule J, *et al.* Task shifting interventions for cardiovascular risk reduction in low-income and middle-income countries: a systematic review of randomised controlled trials. *BMJ Open* 2014;**4**:e005983.

19 Joshi R, Alim M, Kengne AP, *et al.* Task shifting for non-communicable disease management in low and middle income countries--a systematic review. *PLoS One* 2014;**9**:e103754.

20 Ministry of Health & Family Welfare G of I. Rural Health Statistics 2015. 2015.

21 Anchala R, Kaptoge S, Pant H, *et al.* Evaluation of effectiveness and cost-effectiveness of a clinical decision support system in managing hypertension in resource constrained primary health care settings: results from a cluster randomized trial. *J Am Heart Assoc* 2015;**4**:e001213.

22 Tricco AC, Ivers NM, Grimshaw JM, *et al.* Effectiveness of quality improvement strategies on the management of diabetes: a systematic review and meta-analysis. *Lancet* 2012;**379**:2252–61.

23 Nair M, Ali MK, Ajay VS, *et al.* CARRS Surveillance study: design and methods to assess burdens from multiple perspectives. *BMC Public Health* 2012;**12**:701.

24 Hayes RJ, Bennett S. Simple sample size calculation for cluster-randomized trials. *Int J Epidemiol* 1999;**28**:319–26.

25 Glasgow R, Vogt T, Boles S. Evaluating the public health impact of health promotion interventions: the RE-AIM framework. *Am J Public Health* 1999;**89**:1322–7.

26 Drummond MF, Sculpher MJ, Torrance GW, *et al. Methods for the economic evaluation of health care programmes*. 2005.

27 Gilks WR, Richardson S, Spiegelhalter DJ. Markov Chain Monte Carlo in Practice. Technometrics. 1996;**39**:512.

28 Stone PW, Chapman RH, Sandberg EA, *et al.* Measuring costs in cost-utility analyses. Variations in the literature. *Int J Technol Assess Health Care* 2000;**16**:111–24.

29 Shillcutt SD, Walker DG, Goodman CA, *et al.* Cost effectiveness in low- and middle-income

For peer review only - http://bmjopen.bmj.com/site/about/guidelines.xhtml

countries: a review of the debates surrounding decision rules. *Pharmacoeconomics* 2009;**27**:903–17.

30 Ali MK, Singh K, Kondal D, *et al.* Effectiveness of a Multicomponent Quality Improvement Strategy to Improve Achievement of Diabetes Care Goals: A Randomized, Controlled Trial. *Ann Intern Med* 2016;**165**:399–408.

Figure 1 – Trial flowchart

For peer review only

For peer review only - http://bmjopen.bmj.com/site/about/guidelines.xhtml

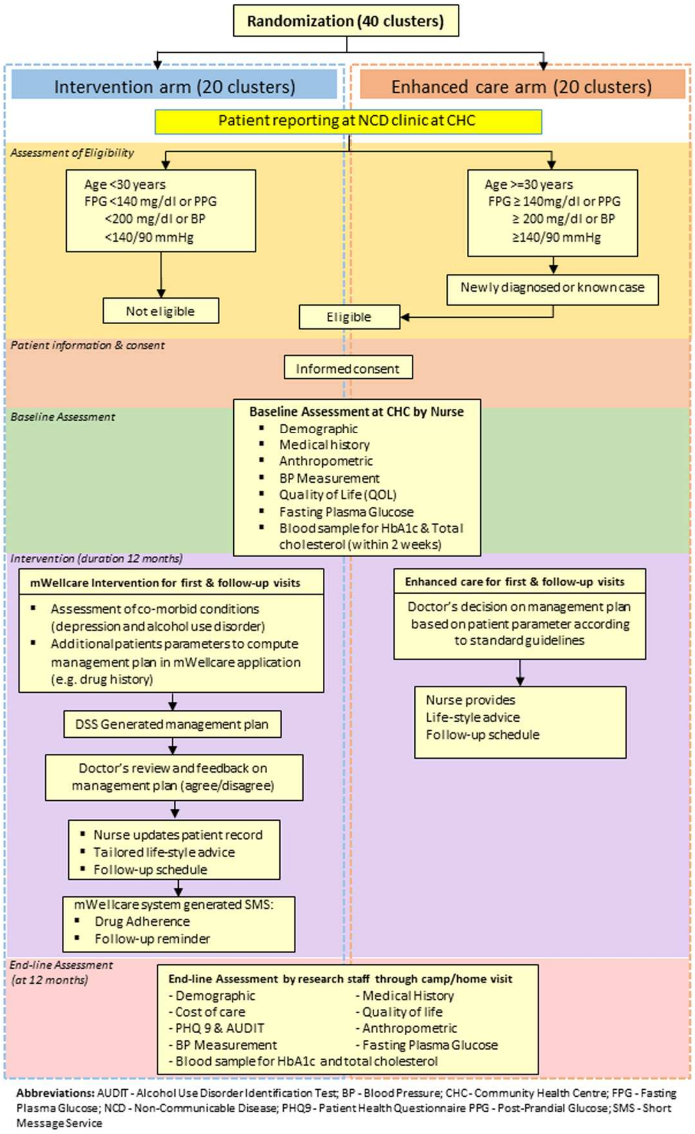

Figure 1 - Trial flowchart

275x397mm (300 x 300 DPI)

SPIRIT 2013 Checklist for mWellcare Trial Protocol

Section	Item No.	Description	Page No. / location in the manuscript
**Administrative Information**			
Title	1	Descriptive title identifying the study design, population, interventions, and, if applicable, trial acronym.	1
Trial Registration	2	Trial identifier and registry name. If not yet registered, name of intended registry	4
Protocol version	3	Date and version identifier	4
Funding	4	Sources and types of financial, material, and other support	12
Roles and responsibilities	5a	Names, affiliations, and roles of protocol contributors	12
	5b	Name and contact information for the trial sponsor	4
	5c	Role of study sponsor and funders, if any, in study design; collection, management, analysis, and interpretation of data; writing of the report; and the decision to submit the report for publication, including whether they will have ultimate authority over any of these activities.	12
	5d	Composition, roles, and responsibilities of the coordinating centre, steering committee, endpoint adjudication committee, data management team, and other individuals or groups overseeing the trial, if applicable (see Item 21a for Data Monitoring Committee).	10
**Introduction**			
Background and rationale	6a	Description of research question and justification for undertaking the trial, including summary of relevant studies (published and unpublished) examining benefits and harms for each intervention.	5
	6b	Explanation for choice of comparators.	5
Objectives	7	Specific objectives or hypotheses	5
Trial design	8	Description of trial design including type of trial (e.g., parallel group, crossover, factorial, single group), allocation ratio, and framework (e.g., superiority, equivalence, non-inferiority, exploratory)	5
**Methods: Participants, interventions, and outcomes**			
Study setting	9	Description of study settings (e.g., community clinic, academic hospital) and list of countries where data will be collected. Reference to where list of study sites can be obtained	5
Eligibility criteria	10	Inclusion and exclusion criteria for participants. If applicable, eligibility criteria for study centres and individuals who will perform the interventions (e.g., surgeons, psychotherapists).	6
Interventions	11a	Item 11a: Interventions for each group with sufficient detail to allow replication, including how and when they will be administered.	6

For peer review only

Section	Item No.	Description	Page No. / location in the manuscript
	11b	Criteria for discontinuing or modifying allocated interventions for a given trial participant (e.g., drug dose change in response to harms, participant request, or improving/worsening disease).	NA
	11c	Strategies to improve adherence to intervention protocols, and any procedures for monitoring adherence (e.g., drug tablet return; laboratory tests).	10
	11d	Relevant concomitant care and interventions that are permitted or prohibited during the trial.	NA
Outcomes	12	Primary, secondary, and other outcomes, including the specific measurement variable (e.g., systolic blood pressure), analysis metric (e.g., change from baseline, final value, time to event), method of aggregation (e.g., median, proportion), and time point for each outcome. Explanation of the clinical relevance of chosen efficacy and harm outcomes is strongly recommended.	7
Participant timeline	13	Time schedule of enrolment, interventions (including any run-ins and washouts), assessments, and visits for participants. A schematic diagram is highly recommended	7
Sample size	14	Estimated number of participants needed to achieve study objectives and how it was determined, including clinical and statistical assumptions supporting any sample size calculations	7
Recruitment	15	Strategies for achieving adequate participant enrolment to reach target sample size	7
**Assignment of intervention**			
Allocation	16a	Method of generating the allocation sequence (e.g., computer-generated random numbers), and list of any factors for stratification. To reduce predictability of a random sequence, details of any planned restriction (e.g., blocking) should be provided in a separate document that is unavailable to those who enroll participants or assign interventions.	8
	16b	Mechanism of implementing the allocation sequence (e.g., central telephone; sequentially numbered, opaque, sealed envelopes), describing any steps to conceal the sequence until interventions are assigned.	
Blinding	17a	Who will be blinded after assignment to interventions (e.g., trial participants, care providers, outcome assessors, data analysts), and how	8
	17b	If blinded, circumstances under which unblinding is permissible, and procedure for revealing a participant's allocated intervention during the trial.	

For peer review only

Section	Item No.	Description	Page No. / location in the manuscript
**Data collection, management, and analysis**			
Data collection methods	18a	Plans for assessment and collection of outcome, baseline, and other trial data, including any related processes to promote data quality (e.g., duplicate measurements, training of assessors) and a description of study instruments (e.g., questionnaires, laboratory tests) along with their reliability and validity, if known. Reference to where data collection forms can be found, if not in the protocol	8
	18b	Retention: Plans to promote participant retention and complete follow-up, including list of any outcome data to be collected for participants who discontinue or deviate from intervention protocols	8
Data management	19	Plans for data entry, coding, security, and storage, including any related processes to promote data quality (e.g., double data entry; range checks for data values). Reference to where details of data management procedures can be found, if not in the protocol	8
Statistical Methods	20a	Outcomes: Statistical methods for analysing primary and secondary outcomes. Reference to where other details of the statistical analysis plan can be found, if not in the protocol.	9
	20b	Additional analyses: Methods for any additional analyses (e.g., subgroup and adjusted analyses).	NA
	20c	Analysis population and missing data: Definition of analysis population relating to protocol non-adherence (e.g., as randomised analysis), and any statistical methods to handle missing data (e.g., multiple imputation)	NA
**Data Monitoring**			
Data monitoring	21a	Formal committee: Composition of data monitoring committee (DMC); summary of its role and reporting structure; statement of whether it is independent from the sponsor and competing interests; and reference to where further details about its charter can be found, if not in the protocol. Alternatively, an explanation of why a DMC is not needed	10
	21b	Interim analysis: Description of any interim analyses and stopping guidelines, including who will have access to these interim results and make the final decision to terminate the trial.	10
Harms	22	Plans for collecting, assessing, reporting, and managing solicited and spontaneously reported adverse events and other unintended effects of trial interventions or trial	10

For peer review only

Section	Item No.	Description	Page No. / location in the manuscript
		conduct.	
Auditing	23	Frequency and procedures for auditing trial conduct, if any, and whether the process will be independent from investigators and the sponsor.	8 &9
**Ethics and dissemination**			
Research ethics approval	24	Plans for seeking research ethics committee/institutional review board (REC/IRB) approval.	12
Protocol Amendment	25	Plans for communicating important protocol modifications (e.g., changes to eligibility criteria, outcomes, analyses) to relevant parties (e.g., investigators, REC/IRBs, trial participants, trial registries, journals, regulators).	NA
Consent	26a	Who will obtain informed consent or assent from potential trial participants or authorised surrogates, and how (see Item 32).	8
	26b	Ancillary studies: Additional consent provisions for collection and use of participant data and biological specimens in ancillary studies, if applicable	NA
Confidentiality	27	How personal information about potential and enrolled participants will be collected, shared, and maintained in order to protect confidentiality before, during, and after the trial.	11 & 12
Declaration of interests	28	Financial and other competing interests for principal investigators for the overall trial and each study site.	12
Access to data	29	Statement of who will have access to the final trial dataset, and disclosure of contractual agreements that limit such access for investigators.	12
Ancillary and post-trial care	30	Provisions, if any, for ancillary and post-trial care, and for compensation to those who suffer harm from trial participation	NA
Dissemination policy	31a	Trial results: Plans for investigators and sponsor to communicate trial results to participants, healthcare professionals, the public, and other relevant groups (e.g., via publication, reporting in results databases, or other data sharing arrangements), including any publication restrictions.	12
	31b	Authorship : Authorship eligibility guidelines and any intended use of professional writers	12
	31c	Reproducible Research: Plans, if any, for granting public access to the full protocol, participant-level dataset, and statistical code.	12

For peer review only
